# WhoGEM: an admixture-based prediction machine accurately predicts quantitative functional traits in plants

**DOI:** 10.1186/s13059-019-1697-0

**Published:** 2019-05-28

**Authors:** Laurent Gentzbittel, Cécile Ben, Mélanie Mazurier, Min-Gyoung Shin, Todd Lorenz, Martina Rickauer, Paul Marjoram, Sergey V. Nuzhdin, Tatiana V. Tatarinova

**Affiliations:** 1 EcoLab, Université de Toulouse, CNRS, Avenue de l’Agrobiopole BP 32607, Auzeville-Tolosane, F-31326 Castanet-Tolosan, France; 2 University of Southern California, 1050 Childs Way (USC), Los Angeles, CA 90089-0371 USA; 3 University of La Verne, 1950 3rd Street, La Verne, CA 91750 USA; 4 Department of Fundamental Biology and Biotechnology, Siberian Federal University, 660074 Krasnoyarsk, Russia

**Keywords:** Genomic prediction, Molecular ecology, Adaptation, Quantitative disease resistance, Breeding, *Medicago truncatula*

## Abstract

The explosive growth of genomic data provides an opportunity to make increased use of sequence variations for phenotype prediction. We have developed a prediction machine for quantitative phenotypes (WhoGEM) that overcomes some of the bottlenecks limiting the current methods. We demonstrated its performance by predicting quantitative disease resistance and quantitative functional traits in the wild model plant species, *Medicago truncatula*, using geographical locations as covariates for admixture analysis. The method’s prediction reliability equals or outperforms all existing algorithms for quantitative phenotype prediction. WhoGEM analysis produces evidence that variation in genome admixture proportions explains most of the phenotypic variation for quantitative phenotypes.

**Electronic supplementary material:**

The online version of this article (10.1186/s13059-019-1697-0) contains supplementary material, which is available to authorized users.

## Background

Living organisms adapt to the changing environment. Species respond to environmental changes by altering population structure via migration, by allele sorting due to random events (genetic drift), and by natural selection [1]. Selective pressure is often imposed by climate conditions, pathogen exposure, food resources, and other variables. Thus, in free-living species, genetics and geography are closely and measurably associated [2]. In most cases, adaptive traits represent measurable phenotypes (i.e., quantitative traits), such as height, yield, fitness, or pathogen resistance, that depend on the cumulative action of many genes with variants occurring across multiple loci, and often poorly understood relationships between loci [3]. In this paper, a large number of mutations of small effect is considered to model the phenotypic effect-size distribution of evolutionary-relevant mutations [4–6]. These evolutionary relevant mutations will likely be key for breeding for complex traits [7], such as fast adaptation to anticipated climate changes in plants or animals, or for predicting carcinogenesis and drug resistance in biomedicine. Small shifts in allele frequencies at many loci may be sufficient to move a phenotype toward some new optimum after a rapid environmental shift [8].

Unlike animals, plants feature complex mating systems including selfing and limited gene dispersal through seeds and pollen and a distinct immune system. Importantly, plants must survive under permanent selective pressure from local environmental conditions. These features make plants excellent subjects to test polygenic adaptation hypotheses and to evaluate the role of migration and drift in the genetic and quantitative phenotypic differentiation among populations [9]. *Medicago truncatula*, a wild legume species, is an attractive model with detailed genomic data available across a number of circum-Mediterranean populations [10–13]. As with other organisms, its current phenotypic and genetic diversity was shaped under the combined action of environment, demography (migration, drift), and mutations. As a self-compatible species, *M. truncatula* is expected to have a more differentiated population structure than outcrossing species [14]. However, contradictory versions of its population structure have been described [15–17].

Many population genetic approaches assume a theoretical framework for the origin of the populations, such as the “stepping stone” [18], hierarchical divergence, or island models [19]. Building on these frameworks, we place our studies of genetic and phenotypic variation into a geographical context. This can provide powerful insights into how historical events, patterns of migration, and natural selection have led to genetic distinctions between various present-day populations [20–22].

The determination of all the genomic variations underlying quantitative traits is challenging [6] and has given rise to a variety of methods. When the adaptive phenotype is not known and is likely to differ among populations, a collection of methods with diverse underlying hypotheses have been implemented. According to the “selective sweep” model [23], the set of variants leading to adaptation is rapidly fixed in the population. This creates a genomic signature which consists of reduced genetic diversity and extended linkage disequilibrium in the genomic region surrounding the loci under selection [24]. Depending on whether a new mutation or standing variation is involved in the adaptive process, hard or soft sweeps may result [25, 26], with recent explorations of whether the majority of sweeps ever go to fixation [27]. In plants, selective sweeps were identified for soil conditions [28] and climate adaptation [29–31]. *F*_*ST*_ scans and other measures of genetic differentiation between population, such as the nucleotide diversity *π* and Tajima’s *D*, similarly allow identification of candidate genes for adaptation [3, 28]. However, polygenic adaptation is difficult to detect using selective sweep tests [32] or via *F*_*ST*_ tests [33] because the spread of selection on a phenotype is distributed over many loci. Gene-environment association methods seek to identify alleles whose frequencies are significantly correlated with environmental variables used as proxies for ecological pressures. Even if the adaptive phenotype is unknown, the correlation analysis suggests the loci that are involved in adaptation [34, 35]. Genetic-environment association methods have increased power to detect selection from standing genetic variation and soft sweeps [36] and may incorporate corrections for populations structure [37, 38]. Also, methods that allow detection of co-varying signals across multiple loci may be useful to detect polygenic adaptation [39], as exemplified by the observed covariance between allelic effects and frequency [40].

When the adaptive phenotype is known or easy to score, a number of experimental approaches aim to identify quantitative trait loci (QTLs) using linkage-based analysis in experimental crosses, or even quantitative trait nucleotides (QTNs) using genome-wide association studies (GWAS). Over recent years, this approach, fueled by increasingly affordable genome sequencing or genotyping, has led to an explosion of disease-related gene discoveries in humans [41]. In plants, one of the major motivations for using GWAS is allele mining, i.e., the identification of novel functional variation that can be deployed in cultivar improvement through marker-assisted selection [42]. Some applications of GWAS in natural populations are also reported [43]. These methods have proven useful for the manipulation of large-effect alleles with known association to a molecular marker [44]. However, quantitative traits influenced by many loci of small effect are sometimes not well predicted by QTLs identified via linkage- or GWAS-based approaches [45]. This leads to the “missing heritability” concept [46, 47]. Some scientists have thus adapted the whole-genome prediction method initially proposed by Meuwissen et al. [48, 49]. The goal of genomic selection (GS) is to predict phenotype using the full set of genome-wide SNPs [50, 51]. GS usually does not identify causal loci, but Bayesian methods of GS can potentially detect SNPs with large effects that can be the causative variants. Also called genomic prediction, GS shows excellent performances for livestock breeding and is now being rapidly implemented in plant breeding [52, 53].

Both selection and population history have important influences on the amount and patterns of genetic variation [54]. As a consequence of having different population genetic histories, distinct sub-populations could have differences in allele frequencies for many polymorphisms throughout the genome. If the populations have different overall values for the phenotype, any polymorphisms that differ in frequency between the two populations will be associated with the phenotype, even though they are neither causal nor in strong linkage disequilibrium with causal polymorphisms [55, 56]. Methods that aim to identify causal loci are therefore highly influenced by population structure. Determination of population structure is at the core of methods based on genomic scans, outlier tests, and genome-environment associations [54, 57]. Typically, population structure inflates *p* values in GWAS [56] and is controlled by the use of linear mixed models that fits population structure and relatedness among individuals within the model [58, 59]. Analyzing the influence of population structure in the training and test sets in GS models is currently an active field of research [60, 61].

Here, we propose and test a novel method to explain variation in genetically complex traits using population admixture proportions of *M. truncatula* individuals, an approach we named “WhoGEM.” The overall goal is to predict quantitative phenotypes rather than identify causative variations or infer the relative role of demography and selection in the evolution of quantitative phenotypes. Gene detection is not the purpose of this model. The WhoGEM prediction machine is developed around three key data inputs, data preprocessing (genotypes), the ProvenancePredictor algorithm (geographical coordinates), and phenotypic characterization (quantitative functional traits). A multi-criteria approach is used to determine the optimal number of population subdivisions for the *M. truncatula* species around the Mediterranean Basin, to assign an admixture proportion vector to each accession, and then to characterize/predict quantitative phenotypes. The ProvenancePredictor algorithm was developed to use geographical covariates as an aid to define the optimum number of admixture components. Significantly, the phenotypic characterization results indicate that admixture proportions of populations explain a significant proportion of several key quantitative functional traits and quantitative disease resistances. The resulting admixture components are also significantly associated with major bio-climatic and geographic variables. This demonstration of the WhoGEM prediction machine indicates that it can outperform the current genomic prediction/genomic selection models typically used to infer quantitative phenotypes in plants and animals. We argue that the WhoGEM prediction machine may be extended to breeds of domesticated plant and animal species, or populations of dividing human cells, that all undergo selective pressure and potentially strong genetic drift.

## Results

**Fig. 1:**
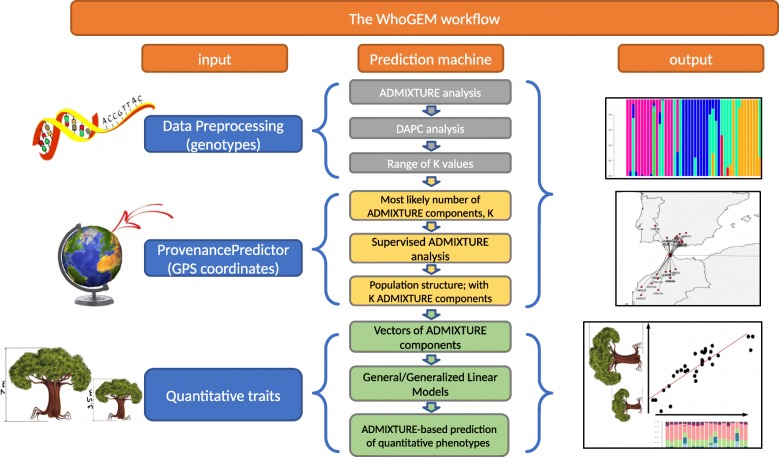
Flowchart of the WhoGEM prediction machine. The inputs (left) include data preprocessing, typically LD-pruned SNPs, the ProvenancePredictor algorithm using geographical locations, and phenotypic characterization. The WhoGEM prediction machine (center) uses geographical information as covariates to help in resolving the number of admixture components. The phenotypic characterization allows predicting quantitative traits using admixture components of individuals. The outputs (right) are covariate-informed population structure and *n*-dimensional admixture vectors, putative localization of unknown samples, and prediction of quantitative traits using admixture proportions

Inferring ancestral genomes that will encounter secondary contact/admixture zones is at the core of the WhoGEM working hypothesis. The most likely number of admixture components representing the putative ancestral genomes was determined using a multi-criteria approach. We conducted a three-step data analysis for the determination of admixture components that (1) defines an initial most likely range of admixture components by minimizing the cross-validation error of admixture [62] analysis; (2) compares this initial guess of admixture components to an independent analysis using the discriminant analysis of principal components (DAPC) method [63], working by optimizing the ratio of the variance between groups to the variance within groups; and (3) checks the accuracy of bio-geographic predictions for various numbers of components using our new ProvenancePredictor algorithm based on the outcome of steps 1 and 2 (Fig. 1).

### Preprocessing: independent methods using LD-pruned SNP data suggest a strong but unresolved population structure for *M. truncatula*

We assessed a collection of 262 *M. truncatula* accessions around the Mediterranean Basin (Additional file 1: Figure S1). Since the ADMIXTURE algorithm requires independence of loci, and to ensure that the DAPC method represents a genome-wide structure and not just reflects the local LD, we first carried out LD pruning leading to a set of 843,171 LD-pruned SNPs covering the 8 chromosomes of the *M. truncatula* genome (Additional file 2: Table S1).

First, using the likelihood-based admixture analysis implemented in ADMIXTURE for *K* = 2 to 12 in the unsupervised mode, we showed that the cross-validation error is minimized for *K* ≥ 7. When considered the admixture plots for *K* = 4...11, we noticed that for *K* = 7 and *K* = 8, the individuals appear homogeneous within their reported regions and distinct between the regions. For *K* > 8, the patterns of admixture component reorganization are inconsistent (Additional file 1: Figure S2).

Second, the range of putative admixture components was compared with the number of groups independently obtained using the discriminant analysis of principal components (DAPC). The set of 843,171 LD-pruned SNPs was submitted to PCA, and 80 principal components were kept to reduce the dimensionality of the data. *K*-means clustering for a range of increasing *K* values, followed by DAPC analysis, was successively performed and assessed using Bayesian information criteria (BIC) criterion. The BIC value was the lowest for *K* = 7 … 9 (Additional file 1: Figure S3), which is in agreement with the values of *K* determined by the ADMIXTURE-based analysis.

### Admixture-based analysis, informed by geographical covariates as implemented in the ProvenancePredictor algorithm, reveals eight admixture components in the *M. truncatula* genome

We used geographical covariates to resolve the number of putative admixture components, using our new ProvenancePredictor algorithm. The ProvenancePredictor algorithm determines the most probable geographical location of a test sample based on its genetic relationships with a geo-localized reference set, by comparing their admixture components. For that, ProvenancePredictor calculated the Euclidean distance between the sample’s admixture proportions and a reference dataset. The shortest distance measure represents the test sample’s genetic deviation from its nearest reference population based on its *n*-dimensional admixture component vector. This admixture-based distance is subsequently converted to geographical distance using the linear relationship observed between genetic and geographic distances (Additional file 7: Code 1). ProvenancePredictor is an adaptation of the admixture-based geographic population structure (GPS) algorithm [64] to plant species. The original GPS algorithm was extensively tested in a number of published studies [65–67]. The modification takes into account ties encountered when the genetic distances between different closely related accessions are estimated as identical given the dataset, a situation that may be encountered with selfing plant species, such as *M. truncatula*, *Arabidopsis thaliana*, or *Oryza sativa* (rice).

The ProvenancePredictor algorithm was thus used to assess the accuracy of geographic assignment for various values of *K*, from *K* = 2 to *K* = 12. The rationale is that the optimal number of admixture components should minimize the distance between observed and predicted locations and maximizes the number of correct assignments of samples to their population of origin. ProvenancePredictor uses the “leave-one-out” cross-validation approach at the “accession” level to estimate the difference between predicted and reported location for each sample (Additional file 8: Code 2). We also calculated the number of correct assignments to the country of origin, as an estimator of population assignment accuracy. The most parsimonious optimum for accurate predictions is achieved for *K* = 8 with 67% of the accessions correctly attributed to their reported country of origin, and 50% of accessions have their location predicted to within 71 km of their recorded location (Additional file 1: Figure S4).

For all subsequent analyses, the 840 K genotype dataset was converted into *K* = 8 dimensional admixture vectors for each accession, determined using ADMIXTURE in the supervised mode. The matrix of pairwise genetic distances was computed using admixture component proportions of each accession. The Mantel test applied to the initial geographical and genetic distance matrices revealed a modest, but nevertheless significant, correlation between geographical and genetic distances (*r* = 0.294, *p* = 1 × 10^−4^). A linear relationship between geographical and genetic distances is restricted to distances less than 950 km (Additional file 1: Figure S5). When filtering out the distance matrices for distances more than 950 km, the Mantel correlation coefficient raises to 0.78, a highly significant value (*p* = 1 × 10^−4^). Thus, a linear relationship between geographical and genetic distances was fitted for geographical distances less than 950 km. The regression equation is *Geo* = 0.204 + 4.973 × Gen + *ϵ* with adjusted *R*^2^ = 0.61 and model *p* < 2.2 × 10^−16^.

### *M. truncatula* has an intricate spatial pattern and population structure around the Mediterranean Basin

**Fig. 2:**
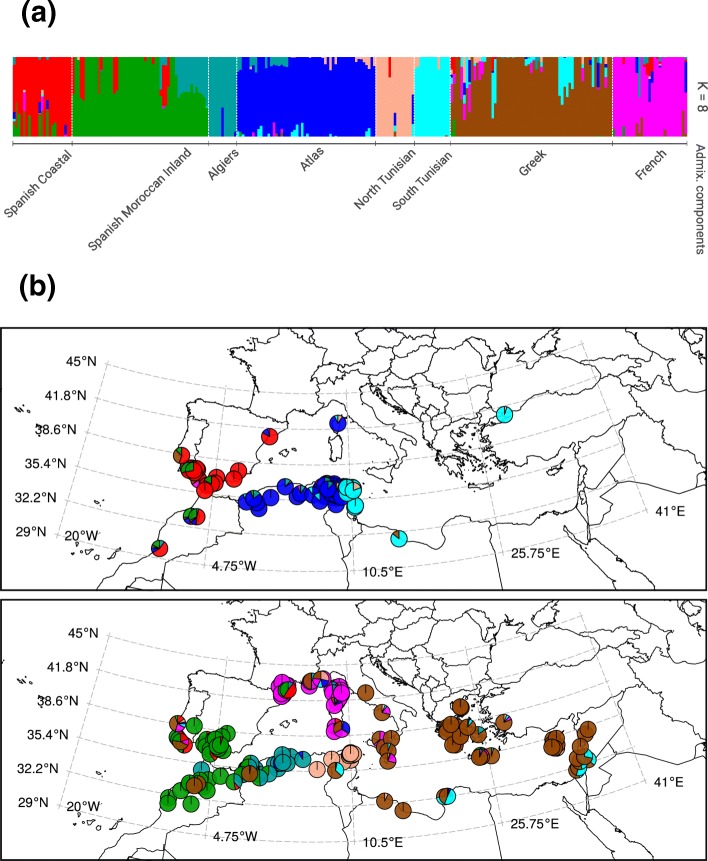
Analysis of 840,171 LD-pruned SNPs reveals patterns of population structure of 262 *M. truncatula* accessions and their geographical distribution around the Mediterranean Basin. **a** The stratification of the collection is obtained assuming *K* = 8. The *x*-axis represents the accessions broadly sorted according to their reported longitude and ancestry. Each accession is represented by a vertical stacked column of color-coded admixture proportions that reflects the genetic contributions from putative ancestral populations. **b** Geographical location of 245 accessions. At each location, a pie chart represents the admixture proportions of the accessions’ genome. Color coding of admixture components is the same as in **a**. A Lambert Conic Conformal Projection (EPSG:3034), suitable for the Mediterranean Basin, was used to draw the geographical maps

This three-step analysis demonstrates that population structure in *M. truncatula* can be adequately explained using eight admixture components (Fig. 2a). We therefore used *K* = 8 components corresponding to eight putative ancestral populations (Additional file 3: Table S2). The name of each population is determined by the region which is the geographical centroid of the accessions of that population. The pair-wise Wright’s *F*_*ST*_ divergences [68] between the admixture components for *K* = 8 (comparing the variance in allele frequencies among the components) indicated that they are strongly differentiated (Table 1a). Figure 2b displays the distribution of the eight putative *M. truncatula* ancestral populations around the Mediterranean Basin, showing the genome admixture proportions of the plant samples. Based on this picture, we assign each population to a representative geographical region (Table 1b). Estimates of *F*_*IS*_ values (the inbreeding coefficient of an individual relative to its sub-population) are similar among the eight populations, suggesting no obvious intra-population heterogeneity (Table 1b). All populations are clearly differentiated, even over short geographical distances, such as with the two Spanish populations.

**Table 1:** Putative ancestral genomes identified by admixture analysis and populations participating to actual levels of structure in *M. truncatula.* (a) Pair-wise *F*_*ST*_ divergences between *K* = 8 admixture components. (b) Characteristics of the eight populations defined using the *K* = 8 admixture components. *F*_*IS*_ fixation index, number of accessions per population, name of the population, and main spanned countries are indicated for each population

(a)							
	K1	K2	K3	K4	K5	K6	K7
K2	0.262						
K3	0.274	0.294					
K4	0.226	0.249	0.105				
K5	0.280	0.296	0.150	0.118			
K6	0.218	0.231	0.146	0.101	0.146		
K7	0.228	0.255	0.127	0.086	0.122	0.086	
K8	0.262	0.229	0.318	0.272	0.322	0.259	0.279
(b)							
Admixture component	*F*_*IS*_	Pop. size	Population name	Country			
K1	0.53	11	Algiers	Algeria			
K2	0.69	23	Spanish Coastal	Spain, Portugal			
K3	0.54	15	North Tunisian Coastal	Tunisia			
K4	0.56	54	Atlas	Algeria, Tunisia			
K5	0.48	13	South Tunisian Coastal	Tunisia			
K6	0.62	29	French	France			
K7	0.58	63	Greek	Greece and neighboring countries			
K8	0.62	53	Spanish-Moroccan Inland	Spain, Morocco			

The divergences among accessions were computed based on the 840 K SNP dataset to estimate relationships among the ancestral populations. The resulting dendrogram (Additional file 1: Figure S6) showed two main clades corresponding to the major divergence event. Clade 1 contains populations from the south-west of the Mediterranean Basin: “Algiers” (K1), “Spanish Coastal” (K2), and “Spanish Morocco Inland” (K8). Clade 2 contains accessions from the north-east of the Mediterranean Basin. For *M. truncatula*, we build on the glacial refugia hypothesis [69] that probably shaped the geographical distributions and patterns of genetic variation of many plant and animal species around the Mediterranean Basin [70]. The data suggests that divergence of clade 1 and clade 2 reflects expansion from glacial refugia during the early Holocene. Within clade 2, the “French” (K6) population is clearly separated from the “Greek” (K7) one, which is in agreement with the “Maritim and Ligurian Alps” glacial refugia hypothesis [71]. Thus, it is conceivable that the initial founders of *M. truncatula* diverged over a large area during glacial and inter-glacial periods, adapted to differing conditions, multiplied in numbers, and then encountered secondary contact/admixture zones starting at the end of the last ice age.

### Geographic localization of the reference genome of *M. truncatula*: ProvenancePredictor confirms that genetics helps predict geography

The *M. truncatula* Jemalong-A17 accession is at the core of a large number of genomic and genetics resources that have been used to study responses to biotic and abiotic stresses and the genetics of symbiotic nitrogen fixation [13, 72]. The Jemalong-A17 accession has been isolated from the Australian Jemalong cultivar (T. Huguet, personal communication); however, the origin of the Jemalong cultivar in the Mediterranean Basin is not documented. Recalling that for a given sample, ProvenancePredictor algorithm determines its provenance (geographical location) where plants with similar genotypes are likely to grow. Therefore, the ProvenancePredictor algorithm was used to infer the geographic source for the *M. truncatula* Jemalong-A17 reference genome [11].

**Fig. 3:**
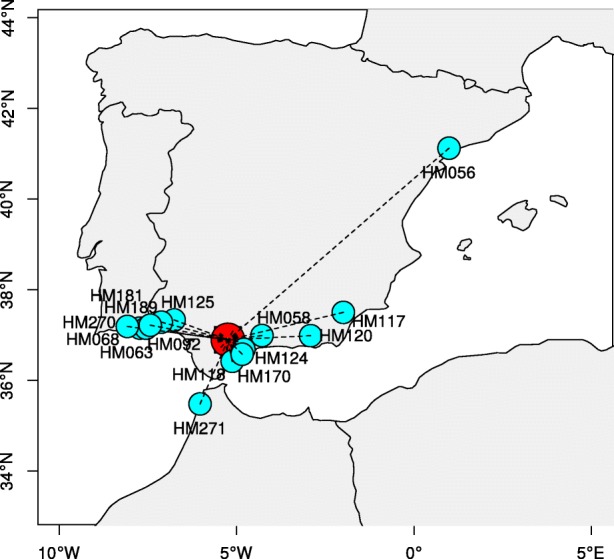
Predicted geographical location of the *M. truncatula* reference accession Jemalong-A17, using the ProvenancePredictor algorithm. The predicted location (in red) is the centroid of the closest accessions, weighted by their genetic distance to Jemalong-A17. Closest accessions with reported geographical location are displayed in cyan

ProvenancePredictor determined that a likely primary geographical position of the Jemalong-A17 be within the “Spanish Coastal” population (Fig. 3). The localization of the Jemalong-A17 reference accession will help to understand its phenotypic characteristics and its responses to stresses. This simple exercise shows the potential of ProvenancePredictor in locating unknown plant samples based on their admixture components and might have similar applications in forensic sciences and technologies.

### Phenotypic characterization using genome admixture components as significant predictors of quantitative functional traits in plants

We investigated whether the *M. truncatula* population structure, as represented by admixture components, might be related to the adaptation for polygenic traits (Fig. 1). The eight-dimensional vector of admixture components is a synthetic representation integrating the effects of gene flow and genetic drift and of natural selection toward local adaptation. It is used as the “whole-genome” model. As a first implementation, the relationships between genome components and phenotypes were estimated using linear models.

### Genome admixture components are predictors of plants’ functional traits

Relationships between admixture component proportions and several quantitative functional traits related to plant development as reported by Stanton-Geddes et al. [73] were assessed.

Additional file 1: Figure S7 depicts the geographical structure of phenotypic values for several nodulation parameters, height, and leaf number combined with admixture proportions of the recorded accessions. Plant height (Table 2a) and number of leaves (Table 2b) exhibit different results regarding the association with genome admixture components. Influence of population structure on plant height is very significant (*r*^2^ = 0.21, *p* = 2 × 10^−11^), but less on the number of leaves (*r*^2^ = 0.05, *p* = 7 × 10^−4^). The results suggest that a latitudinal cline for leaf numbers may exist, with accessions south of the Mediterranean Basin harboring more leaves. For nodulation efficiencies, key adaptive traits for legume plants, we describe very significant relationships between genome admixture components and two nodulation parameters (*r*^2^ = 0.10 and *r*^2^ = 0.15 for a total number of nodules and number of nodules in the top 5 cm of the root; Table 2c to Table 2e).

**Table 2:** Admixture components allow predicting several quantitative functional traits in *M. truncatula*. (a) Linear model between admixture components and final plant height before harvest. (b) Linear model between admixture components and number of leaves at about 2 weeks. (c) Linear model between admixture components and number of nodules below 5 cm of root growth. (d) Linear model between admixture components and number of nodules in top 5 cm of roots. (e) Linear model between admixture components and total number of nodules. Raw data from Stanton-Geddes et al. [73]

	Estimate	Std. error	*t* value	Pr (. > |*t*|)
(a)				
Intercept	14.1608	0.3506	40.39	0.0000
South Tunisian Coastal	5.7582	1.1345	5.08	0.0000
Greek	3.6659	0.6954	5.27	0.0000
North Tunisian Coastal	5.0274	1.1228	4.48	0.0000
Spanish Coastal	3.7344	0.9770	3.82	0.0002
*r*^2^ = 0.21. *P* = 1.5 × 10^−11^				
(b)				
Intercept	2.8234	0.0444	63.54	0.0000
French	− 0.4447	0.1482	− 3.00	0.0030
Atlas	0.1995	0.1007	1.98	0.0488
*r*^2^ = 0.05. *P* = 7.3 × 10^−4^				
(c)				
Intercept	14.7805	0.5198	28.44	0.0000
Spanish Coastal	5.0685	1.7406	2.91	0.0040
South Tunisian Coastal	− 4.9235	2.0711	− 2.38	0.0183
*r*^2^ = 0.06. *P* = 4.2 × 10^−4^				
(d)				
Intercept	5.1755	0.2108	24.55	0.0000
South Tunisian Coastal	− 2.4748	0.7870	− 3.14	0.0019
Spanish Coastal	2.6320	0.6618	3.98	0.0001
Algiers	3.1375	0.8330	3.77	0.0002
*r*^2^ = 0.15. *P* = 1.1 × 10^−8^				
(e)				
Intercept	19.7448	0.6824	28.93	0.0000
Spanish Coastal	7.9483	2.1422	3.71	0.0003
South Tunisian Coastal	− 7.1137	2.5473	− 2.79	0.0057
Algiers	5.3812	2.6964	2.00	0.0472
*r*^2^ = 0.10. *P* = 6 × 10^−6^				

### Cross-validation estimates how accurately admixture-based predictive models will perform in practice

The WhoGEM approach is akin to the calculation of phenotypic resemblance as in genomic selection/genomic prediction methodology [48, 49] that uses genome-wide SNP information to enhance predictive ability. Thus, the WhoGEM metric was compared to five genomic selection/prediction methods, namely ridge regression best linear unbiased predictor (RR-BLUP) and kinship-BLUP (G-BLUP) [74], BayesB [48], reproducing kernel Hilbert space (RKHS) [75], and least absolute shrinkage and selection operator (LASSO) regression [76], for associating genotype to phenotype in the 262 entries.

In this work, repeated *k*-fold cross-validation is used to evaluate and compare the models. It is a robust, nonparametric technique that is assumption-free and comparable across models. The method consists of splitting the data *y* into a training data set (*y*_1_) and a validation data set (*y*_2_), given some putative constraints, such as population structure or spatial proximity. Model parameters are estimated in the training data set. Parameter estimates from *y*_1_ are then used to predict observations in the validation data set (i.e., ${\hat{y}}_2\mid {y}_1$). A function relating the predicted and true observations summarizes the performance of the model. Pearson’s correlation was used among predicted (${\hat{y}}_2$) and realized observations (*y*_2_) in the data set to test the reliability of the models. The reliability is proportional to the phenotypic variation explained by the models [77]. We set up a repeated *k*-fold cross-validation based on 50 rounds of fivefold cross-validation. For each fold, proportional sampling of the training set in the eight *M. truncatula* populations is conducted to include the constraint due to the population structure and to be as close as possible of realized observations.

**Fig. 4:**
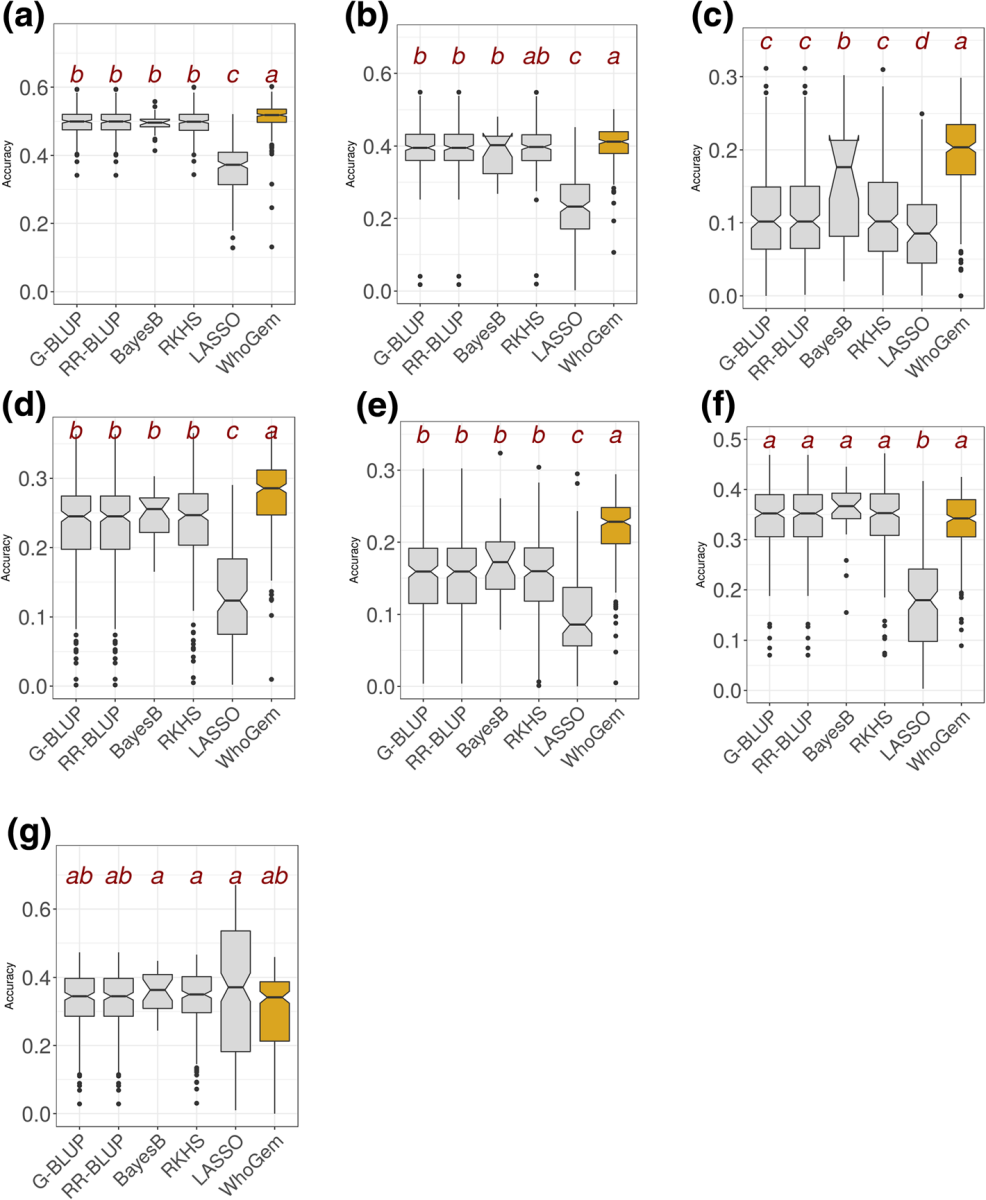
Reliability of five genomic prediction algorithms (G-BLUP, RR-BLUP, BayesB, RKHS, LASSO) and of WhoGEM method to predict quantitative traits in *M. truncatula.* Reliability is estimated using 50 rounds of fivefold cross-validation (repeated *k*-fold cross-validation). **a** Maximum symptom score for the response to *Verticillium alfalfae*. **b** Plant height. **c** Number of leaves. **d** Total number of nodules. **e** Number of nodules below 5 cm of the root. **f** Number of nodules above 5 cm of the root. **g** Root rot index for the response to *Aphanomyces euteiches*. If the notches of two boxes do not overlap, this suggests that the medians are significantly different. Letters identify significantly different groups, with Kruskal-Wallis rank test at *α* = 0.01

Figure 4 summarizes the comparisons of the reliability of predictions of quantitative traits using WhoGEM with predictions by five major algorithms used for genomic selection. These results indicate that the WhoGEM prediction machine outperforms the GS algorithms for traits with low heritability [73] and with low reliability of prediction by GS. This is the case for the number of leaves (Fig. 4c), for the total number of nodules (Fig. 4d), or number of nodules below 5 cm of the root (Fig. 4e). The WhoGEM prediction machine performs as good as the GS algorithms for traits such as plant height (Fig. 4b) or the number of nodules above 5 cm (Fig. 4f).

### Genome admixture components are predictors of the most common form of disease resistance in plants: quantitative disease resistance

Knowledge of the selective pressure acting on the phenotype can help determine the contributions of adaptive selection and drift toward phenotypic differentiation among populations. Consequently, by comparing the location of plants and testing for pathogen resistance, the WhoGEM analysis facilitates a better understanding of phenotypic traits associated with quantitative disease resistance (QDR). Two types of disease resistance are described in plants: (i) complete resistance conditioned by a single gene [78] and (ii) partial resistance, also called QDR, conditioned by multiple genes of partial effect [79]. QDR often confers broad-spectrum resistance, being predicted to be critical for efficient control of epidemics. It is characterized by a continuous range of phenotypes from susceptible to fully resistant. QDR is often described by QTL that supports the resistant phenotype and suggests modes of polygenic adaptation [79]. Studies that attempt to dissect a QDR trait have reported genes with various biological functions such as ABC transporters [80] or atypical kinases [81]. However, these genes do not explain all genetic variability reported in controlled crosses or GWAS studies. We tested the WhoGEM prediction machine to evaluate the proportion of quantitative resistance to two diseases explained by admixture components (Fig. 1).

**Fig. 5:**
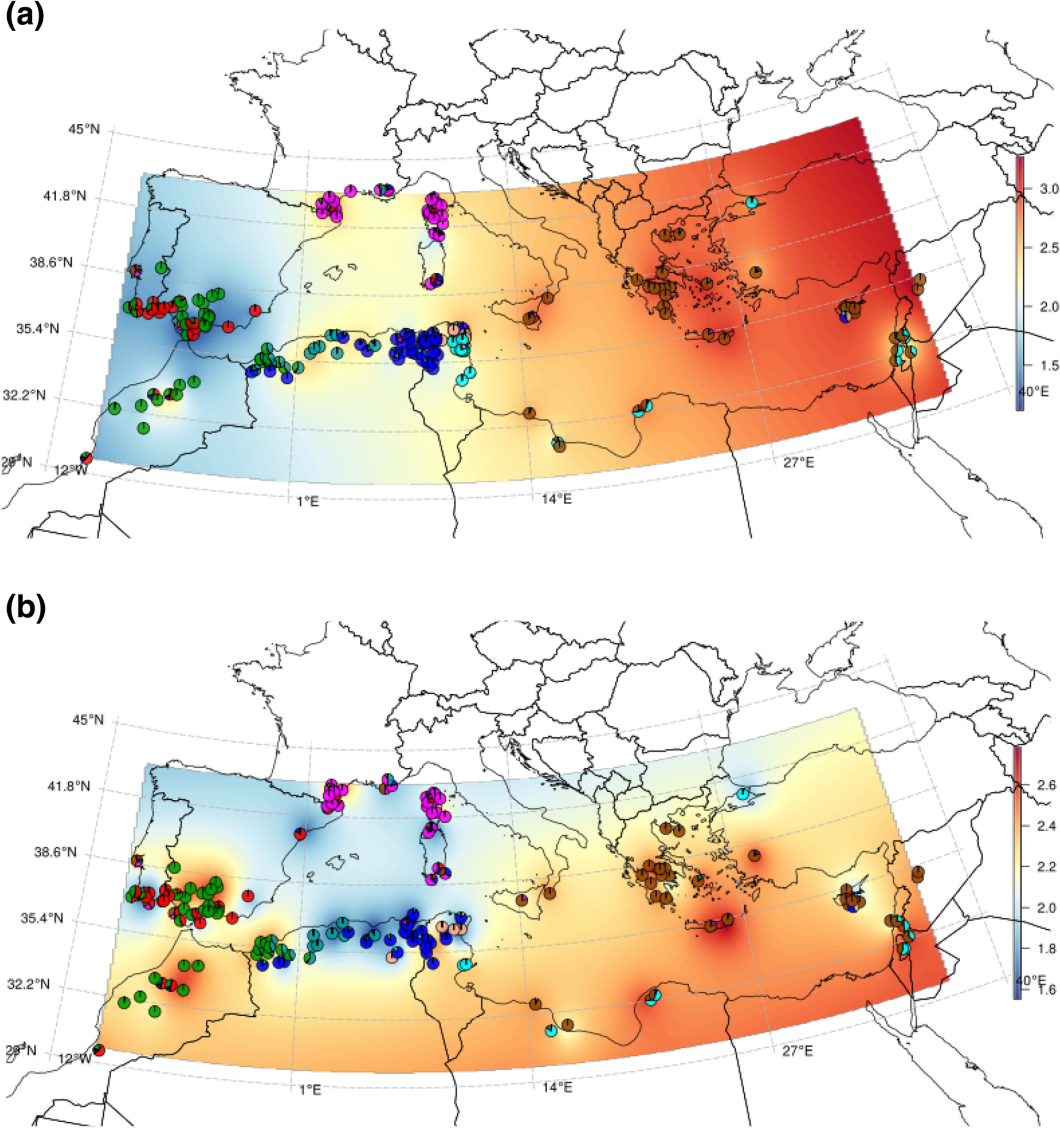
Geographical repartition of quantitative disease resistances to soil-borne root pathogens in *M. truncatula.* **a** Maximum symptom score (MSS) in response to *V. alfalfae*, in a collection of 242 accessions. The MSS scale is displayed as a color gradient. The scale of MSS from resistant (blue) to susceptible (red) accessions is indicated on the right. Admixture proportions of each phenotyped accession are summarized by pie charts. **b** Root rot index following infection of 174 accessions by *A. euteiches*. The RRI scale is displayed as a color gradient. The scale of the index from resistant (blue) to susceptible (red) accessions is indicated on the right. At each evaluated accession, a pie chart presents the admixture patterns. Raw data from Bonhomme et al. [17]. A Lambert Conic Conformal Projection (EPSG:3034), suitable for the Mediterranean Basin, was used to draw the geographical maps

*M. truncatula* is prone to infection by the soil-borne fungal vascular pathogen *Verticillium alfalfae*. Verticillium wilt response in *M. truncatula* is a QDR, regulated by QTLs that differ across resistant accessions and vary according to the fungal strains [82, 83]. Both plant and fungal species co-exist around the Mediterranean Basin (CABI database, PlantWise database http://www.plantwise.org/, accessed on October 19, 2017). Figure 5a shows the geographical partition of the maximum symptom score (MSS) of 242 *M. truncatula* accessions when infected with the *V. alfalfae* strain V31–2 (Additional file 4: Table S3), together with their admixture patterns. Accessions located west of the Mediterranean Basin are mainly resistant to the V31–2 strain (low MSS), while accessions located east of the Mediterranean Basin are susceptible (high MSS). An independent phenotypic evaluation of 32 other accessions picked randomly from the “Spanish Coastal” or “Spanish-Moroccan” geographic zone and of 39 other accessions picked from the “Greek” geographic zone (Additional file 1: Figure S8a) confirms these results and excludes the possibility of a sampling bias (Additional file 1: Figure S8b, Additional file 5: Table S4, and Additional file 6: Table S5).

The findings (Table 3a) show that the values of four admixture components are significantly related to MSS (*r*^2^ = 0.31, *p* ≤ 2.2 × 10^−16^). The average MSS values of the “Spanish Coastal,” “Spanish-Moroccan Inland,” and “South Tunisian Coastal” genome components were found to be 1.04, 1.6, and 1.71, respectively, indicating resistant genomic backgrounds. The average MSS value of the “Greek” genome component is ≃3, making it a clearly susceptible genomic background. Interestingly, the predicted location of Jemalong-A17 within the “Spanish Coastal” population agrees with its resistant phenotype in response to *V. alfalfae* [84]. We have, therefore, made predictions of the QDR level in *M. truncatula* using WhoGEM. The phenotypic difference between predicted resistant and susceptible accessions was around two points on a scale from 0 to 4, i.e., 50% of the phenotypic difference between extremes of the phenotype distribution. Given the estimated narrow sense heritability of the trait [82, 83], we suggest that genome admixture components explain most of the genetic control of this disease. Figure 4a shows that the reliability of the prediction of QDR to *V. alfalfae* by the WhoGEM prediction machine outperforms the five major algorithms used for GS analyses (Kruskal-Wallis *χ*^2^ = 510.37 for 5 degrees of freedom, *p* = 2.2 × 10^−16^).

**Table 3:** Admixture proportions allow predicting quantitative disease resistance to soil-borne pathogens in *M. truncatula*. (a) Linear model between admixture components and maximum symptom scores in response to *Verticillium alfalfae* in a collection of 242 accessions. (b) Linear model between admixture components and root rot index due to infection by *Aphanomyces euteiches*. Data from Bonhomme et al. [17]

	Estimate	Std. error	*t* value	Pr (. > |*t*|)
(a)				
Intercept	2.4541	0.0861	28.50	0.0000
Spanish Coastal	− 1.4147	0.2290	− 6.18	0.0000
South Tunisian Coastal	− 0.7222	0.2367	− 3.05	0.0026
Greek	0.6017	0.1636	3.68	0.0003
Spanish-Moroccan Inland	− 0.8518	0.1804	− 4.72	0.0000
*r*^2^ = 0.31. *P* = 2.2 × 10^−16^				
(b)				
Intercept	2.7362	0.1237	22.12	0.0000
Algiers	− 1.2335	0.2816	− 4.38	0.0000
Spanish Coastal	− 1.1635	0.2353	− 4.95	0.0000
North Tunisian Coastal	− 1.4798	0.3648	− 4.06	0.0001
Atlas	− 0.7511	0.1918	− 3.92	0.0001
French	− 0.5753	0.1963	− 2.93	0.0038
Greek	− 0.3084	0.1812	− 1.70	0.0906
*r*^2^ = 0.19. *P* = 1.8 × 10^−7^				

The oomycete *Aphanomyces euteiches* is another soil-borne pathogen of legume crops, mainly occurring north of the 45th parallel. Two closely linked major loci for resistance to *A. euteiches* root rot were reported by GWAS, which explain 23% of the genetic variance [17]. Using these reported data, we analyzed the geographical structure of the root rot index (RRI) with the admixture patterns of the studied accessions (Fig. 5b). RRI is a typical phenotype for evaluating resistance. Testing whether the proportions of admixture components (Additional file 3: Table S2) were predictors for RRI, we found a significant relationship between the values (Table 3b). Admixture components from the “Algiers,” “Spanish Coastal,” “North Tunisian Coastal,” and “Atlas” populations provide resistance alleles, whereas components from the “Greek” and “French” populations provide susceptibility alleles. The WhoGEM model accounted for *r*^2^ = 19.2 % of the variation in the phenotype and may provide a lower bound for heritability. Figure 4g shows that the reliability of the prediction of the root rot index by WhoGEM and other GS algorithms is similar (“ab” group of means). For this particular trait, the LASSO algorithm is performing slightly better (“a” group of means). According to Tibshirani (Tibshirani 1996), LASSO, that is a variable selection method, would perform better than other methods on a dataset with a small portion of variables having large effects and the others with negligible effects [85]. It is likely that QDR toward *A. euteiches* is controlled by a few major loci, because it was ascribed to two closely linked major loci [17]. This may provide a rationale to understand the better performance of LASSO compared to all other algorithms for that particular genetic architecture.

### Variations in admixture proportions are significantly correlated with geographical and bioclimatic variables that explain a large part of genetic variation in *M. truncatula*

Controlling for population structure may limit the power to detect true adaptive polymorphisms that are collinear with population structure [86], as evidenced by Lasky et al. [34]. Admixture components, integrating demography and natural selection, would be useful tools to test for genetic-environment associations.

**Fig. 6:**
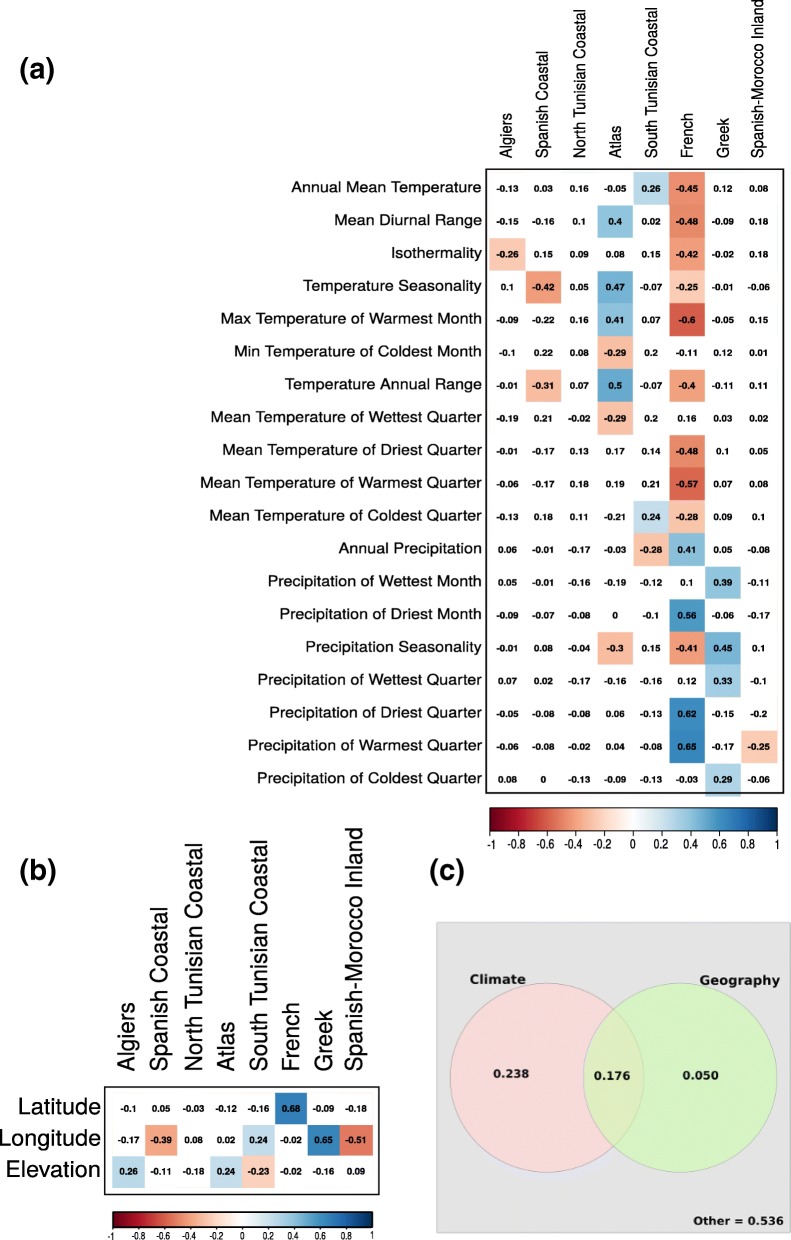
Relationships between the 8 admixture components in 262 *M. truncatula.* Accessions and environmental variables. **a** Pearson correlation coefficient with geographical coordinates. Significant correlations (*p* value 0.01, with Bonferroni correction) are colored. **b** Pearson correlation coefficient with 19 bioclimatic variables defined by WorldClim. Significant correlations (*p* value 0.01, with Bonferroni correction) are colored. **c** Venn diagram of the variation partitioning for genome admixture component proportions explained by climate (left) and geography (right). Residual is the amount of genomic variation not explained by the two explanatory variables

Examination of the assignment of eight *M. truncatula* ancestral populations to climatic zones defined by the Köppen-Geiger climate classification [87] suggests that current global climatic types cannot be the only forces shaping *M. truncatula* populations. Different populations are present in the same climatic zone, while the “Greek” population is spread across several climatic zones (Additional file 1: Figure S9). We thus analyzed the associations between admixture components and 19 local bio-climatic variables, defined by WorldClim (http://www.worldclim.org). Pearson’s correlation coefficients between each admixture component and each bio-climatic or geographical variables are shown in Fig. 6. There is a wide range of magnitudes and direction of associations between bio-climatic variables, geographical coordinates, and admixture components. For example, the “Spanish Coastal” component is negatively correlated with the temperature seasonality and temperature annual range, indicating that this genome corresponds to accessions growing in regions with moderate annual temperature and small temperature seasonal contrasts. As a second example, longitudinal east-west gradients for proportions of Greek genome and both Spanish genomes are evidenced. Interestingly, the admixture proportions of the “North Tunisian Coastal” population are not correlated with any bio-climatic variable, suggesting that the differentiation of this genome may be due to other factors. Friesen et al. [88] described how accessions belonging to this population harbor alleles that assort non-randomly with soil salinity, suggesting a differentiation of the “North Tunisian Coastal” population arose due to this particular abiotic condition.

Next, redundancy analysis (RDA) [89] was used to partition genomic variation summarized by admixture proportions into components explained by climate and geography. RDA examines how well of variation in one set of variables (bio-climatic variables and/or geography) explains variation in another set of variables (the eight-component admixture proportion vector of each sample). As such, RDA allows estimating the change in the structure of genomic variation across spatial scales (latitude, longitude, and elevation) and climatic variables. Figure 6c shows approximately half of the genomic variation is due to climate or geography, separately or in combination (*r*^2^ = 0.46; *P* ≤ 0.001), with climate being the major source of variation (41.4%). Variation explained by geography alone contributes to 5% only. This partition of genomic variation in response to climate is different to *A. thaliana*, in which climate variation among sites of origin explained only slightly more genomic variation than geographical distance [70, 89].

## Discussion

Linking specific genomic variations to selective traits in plants, animals (yield, fitness, etc.), and humans (disease predisposition, drug response, etc.) is a key task for many fields from ecology, plant and animal breeding, to individualized health care and drug discovery. The quantitative phenotypic variability found in natural populations is due to a complex underlying genetic interplay of multiple, often unknown, loci with allelic effects affected by environmental conditions [5, 45, 90]. Similarly, a large number of selected traits in breeds of domesticated species occur via the evolution of quantitative, polygenic traits [25, 91]. In those cases, identifying all the genomic variations underlying these traits is highly challenging [6] and motivated the development of a variety of methods.

WhoGEM is a powerful method that can be used to study natural variation. The method predicts quantitative phenotypes, not focusing on identifying causative variations or inferring the relative parts of demography and selection in the evolution of quantitative phenotypes. The method uses population admixture proportions of individuals to explain variation in genetically complex traits. We explicitly consider admixture proportions to embed population differentiation due to neutral processes such as genetic drift, migration, and mutation [92]. Admixture proportions also reflect the adaptive divergence of ancestral populations at their initial locations, with putative differential introgression depending on the environmental fit [93]. The use of admixture components, that integrate the effects of demography (i.e., gene flow and genetic drift) and of natural selection, thereby explains more phenotypic variation than the current methods. The utility of the WhoGEM prediction machine when inferring complex phenotypes (Fig. 1) is illustrated by extensive performance tests.

The WhoGEM method relies on a thorough inference of ancestral populations that define the admixture components. Finding the most likely number of the ancestral populations and the optimal assignment of the samples to these populations is critical for WhoGEM’s efficiency. Because it often appears that several possible numbers of ancestral populations (*K*) may be in accordance with the dataset, WhoGEM prediction machine will improve the inference of *K* using covariates. In this study, we took advantage of the knowledge of geographical coordinates of the accessions. Comparing the observed geographical locations and those predicted by ProvenancePredictor, we are able to propose a most parsimonious value of the number of admixture components. The admixture component pattern of each sample then provides a comprehensive summary of each genome and is used as linear predictors of quantitative phenotypes.

The use of geographic coordinates is an obvious choice of covariates to help the inference of population structure in wild species. This investigation also demonstrates the potential of the ProvenancePredictor algorithm in locating unknown plant samples based on their admixture components and suggests that it may have similar applications in forensic sciences and technologies. For cultivated plant species as an example, putative geographic locations of original germplasms and characteristic data of current cultivation areas, such as climate variables and soil data, will be critical covariates to integrate. Thus, diverse information may be used as a covariate to help in resolving the most likely number of admixture components. This will require designing appropriate tests to evaluate their pertinence in improving the determination of the number of admixture components.

Our results point to *M. truncatula* ancestral populations probably shaped by glacial refugia around the Mediterranean Basin. The glacial refugia hypothesis assumes that refuge areas of the late Ice Age remained isolated for long periods corresponding to thousands of generations and leading to population differentiation. Gene flow decreased with increasing adaptive divergence in each refugium [93]. *M. truncatula* probably survived the last Ice Age in Iberia, Algeria, the south-east of France, and Greece, and these were likely distinct glacial refugia [71]. After the end of the Ice Age, Greek *M. truncatula* expanded west and south and this unrecombined “Greek” genome has spread uninterrupted over great distances. It is also tempting to speculate that human Greek colonization of the Mediterranean Basin [94] favored the dissemination of the Greek genome. Ellwood et al. [15] already hypothesized that trades and livestock movements may have been causal in this species dispersal. The pods of *M. truncatula* possess spines with hooks that allow them to easily cling to fur or clothing and may help in long distance dispersion. Recent data in *Arabidopsis thaliana* suggest the existence of relict populations from some glacial refugia and the putative importance of humans in the dispersion of this wild species [21, 70]. Glacial refugia in the Maghreb regions may have been more abundant [71], generating more complex patterns of the population in this area. In comparison, Ronfort et al. [16] and latter Bonhomme et al. [17] reported only a faint structure among *M. truncatula* populations, with a major difference between the east and the west of the Mediterranean Basin. Deductions based on the WhoGEM analysis favor the hypothesis of a significant diversity and support previous results that described the differentiation within Tunisian populations similar to the “Atlas” and “North and South Tunisian Coastal” genomes [95].

QDR is typically broad spectrum, making the arms race between hosts and pathogens probably not critical. Our results support the idea that QDR in plants is likely to result from changes to a large number of genes scattered throughout the genome and that this is reflected in admixture proportions. Because of the co-occurrence of both the plant species and the pathogen around the Mediterranean Basin, we hypothesize that the observed pattern of quantitative resistance in the *M. truncatula*/*V. alfalfae* pathosystem may be due to natural selection, with additional contributions from drift and migration. The situation for QDR to *A. euteiches* is different. The populations of the Maghreb area showed a contrasting response to the pathogen. The “Spanish-Moroccan Inland” population is susceptible, compared to the resistant “Algiers” and “Atlas” populations. A zone with admixed accessions exhibiting intermediate phenotypes can be seen in the region of Oran in Algeria where the “Spanish-Moroccan Inland” and “Atlas” populations are in contact (Fig. 5b). Intriguingly, the *A. euteiches* pathogen is not reported in North Africa (CABI database, PlantWise database http://www.plantwise.org/, accessed on October 10, 2018; [17]). Hence, we hypothesize that phenotypic differentiation among resistant and susceptible populations of the Maghreb may be due to either genetic drift or migration. The maintenance of the resistant phenotype in the “Algiers” and “Atlas” populations, where the pathogen is absent, also suggests that the cost of resistance may be negligible in the absence of pathogen, in contrast with previous results described for foliar pathogens [96]. An alternative hypothesis is that resistance to *A. euteiches* is driven by, or in strong LD to, the resistance to other factors, as suggested by Djebali et al. [97], and, as such, not a consequence of natural selection acting toward oomycete resistance. Based on covariate-informed admixture component assessment, the comparative analysis of quantitative disease response to two different pathogens clearly demonstrates that phenotypic differentiation among populations may (in the case of partial resistance to *V. alfalfae*) or may not (in the case of partial resistance to *A. euteiches*) result from natural selection. Plant pathosystems are convenient experimental systems to test for the existence of adaptive divergences among populations, especially when the co-occurrence (or absence thereof) of the plant species and pathogens is known [98]. This advantage is well known and used for the study of plant gene-for-gene resistance [78, 99] and prove to be particularly attractive for QDR. Geographical knowledge of the co-occurrence of plant species and pathogens helps to identify cases where genetic drift or migration play a key role.

Here, we present a methodology for the prediction of quantitative traits using admixture components including covariates, such as geographical origins. This was achieved by converting the large 840 K genotype data into *K* = 8 dimensional vectors for each accession. The vectors represent combinations of genes (either protein-encoding or regulatory, such as non-coding RNAs) manifested as alleles, copy-number variants, and some other genetic or epigenetic variants. These vectors can be considered as “whole-genome” models in providing information integrated across the entire genome. Thus, the WhoGEM prediction machine moves away from focusing on large impact variants [100, 101] or lists of numerous candidate SNPs [102, 103]. Instead, we proposed to calculate a simple descriptor of “mixing proportions” in individuals believed to originate from distinct ancestral populations. The proposed method differs from standard GWAS in that there is no selection of SNPs based upon test statistics for the association between functional traits and SNPs. Consequently, we do not suffer from the “winner’s curse” effect (the systematic overestimation of SNP effects ascertained by thresholding) [104, 105], or from the Beavis effect (L-shaped distribution of effect sizes for SNPs, even when the underlying loci have identical effect sizes) [106]. The WhoGEM approach is akin to the calculation of phenotypic resemblance as in the whole-genome genetic resemblance method of genomic selection/prediction [48, 49], which uses genome-wide SNP information to enhance predictive ability. Unlike the latter approach, WhoGEM explicitly embeds the inferred population structure in the calculations, thus expanding the method’s applicability. Integrating the effects of demography and of natural selection allows predicting more phenotypic variation than current methods, as exemplified by a greater reliability of prediction for low-heritability traits. Moreover, WhoGEM prediction machine considerably simplifies the computations.

As an alternative to the admixture approach, redundancy analysis can be applied to raw SNP data, instead of admixture proportions [35]. Other genetic-environment association methods, such as BAYENV/BAYENV2 [37], BAYPASS [107], or LFMM [36], are able to identify significantly differentiated SNPs. These methods also account for the fact that allele frequencies are correlated among closely related populations. For each studied trait, such strategies may help in identifying if it is either monogenic, oligogenic, or highly polygenic [89], but will not provide a proper benchmark of the WhoGEM approach.

The WhoGEM concept is likely to be expandable to other quantitative functional traits that involve complex genetic determinism. How one would determine whether WhoGEM prediction machine would perform better in a given context is an exciting follow-up topic to develop, in varying the biological models, heritability of traits, and priors on genetic architectures. Prediction of simply inherited traits, not related to population structure, will not be accurate using whole-genome population-based models. In those cases, GWAS analyses are likely to be the most efficient way. We anticipate that an appropriate model to identify major-effect loci for some quantitative traits would be to run mixed effect models where admixture components, as determined here, would be used as fixed co-factors. Moreover, the method we use to improve population structure analyses by using covariates holds strong interest to correct *p* value inflation in GWAS analyses. Furthermore, it will be interesting to use WhoGEM to analyze quantitative phenotypes in breeds of domesticated species, where population structure is often strong due to breeding history [108].

## Conclusions

This study demonstrates the rationale of our WhoGEM prediction machine: population admixture integrates the effects of demography (i.e., gene flow and genetic drift) and of natural selection toward adaptation and thereby explains more phenotypic variation than GS- or QTL-based approaches. The method is thus indifferent to the source of genetic similarity among samples—local adaptation or demographic history. Typically, predicting phenotypes on the basis of genome admixture components will help in inferring future trends of adaptation related to global climate change, where controlling for population structure may limit power to detect true adaptive polymorphisms that would be collinear with current population structure [34, 86]. Finally, prediction of complex traits in humans, for example, drug response in clinical trials or disease predisposition models, may also benefit from the same general methodology. An extension of WhoGEM would be capable of integrating and calculating admixture proportions from multiple types of genome-wide “big data,” such as epigenetics and expression profiling. This approach can also be applied to the analysis of a wide range of bio-medical problems, such as prediction of drug response and carcinogenesis, and can accelerate breeding programs in agriculturally important plants and animals.

## Materials and methods

### SNP selection

A set of 262 genuine *Medicago truncatula* accessions [109] was used to extract SNPs downloaded from (http://www.medicagohapmap.org). Quality checking and LD pruning was done using PLINK [110] with the options –geno 0.05 –maf 0.01 –indep 300 60 1.3.

### Population structure

The strategy used to identify populations combines three steps: admixture-based tools, discriminant analysis of principal components (DAPC), and ProvenancePredictor.

First, we use the ADMIXTURE software package [62] applied to the collection of high-quality LD-pruned SNPs. Each plant sample is characterized by a vector of *n* proportions that sum to one, *n* being the number of admixture components (i.e., *n* = *K*). Computations were conducted independently twice and produced almost identical results. Second, the most suitable number of populations was assessed using discriminant analysis of principal components (DAPC) [63]. DAPC computations were performed using the R package adegenet using VCF-formatted files.

### Development of the ProvenancePredictor algorithm

ProvenancePredictor is an adaptation of the admixture-based geographic population structure (GPS) algorithm [64] to plant species. The matrix of admixture proportions was calculated with the ADMIXTURE software package. The shortest distance measure was converted to geographical distance using the linear relationships observed between genetic and geographic distances (see below). The final position of the sample on the map was calculated by a linear combination of vectors, with the origin at the geographic center of the best matching population weighted by the distances to 10 nearest reference populations and further scaled to fit on a circle with a radius proportional to the geographical distance. If the smallest distance (${\Delta}_{\mathrm{GEN}}^{\mathrm{min}}$) that represented the sample’s deviation from the best matching accession was identical for several accessions, those were considered as ties and included in a single set. Numerical values therefore may contain ties, and the geographical position of an unknown accession was defined as the centroid of the geographical positions of the identical, or nearest accessions. The contribution of other reference accessions *m* = 2. . *N* to the sample’s genetic make-up might also contain ties. The computation of the weight $w=\frac{\Delta_{\mathrm{GEN}}^{\mathrm{min}}}{\Delta_{\mathrm{GEN}}(m)}$ was then modified accordingly.

To convert genetic distance based on admixture proportions to geographical distance, the correlation between geographic and genetic distances between pairs of individuals was estimated for each value of *K* and a linear model fitted. Given the (relatively) small distances across the Mediterranean Basin, we computed a “naive” geographical distance using pairwise Euclidean distance based on the longitude/latitude reported for the accessions.

To estimate the assignment accuracy of ProvenancePredictor, we used the “leave-one-out” approach at the individual level. In brief, we excluded each reference individual from the data set, recalculated the mean admixture proportions of its reference population, predicted its biogeography, computed the geographical distance between predicted and reported locations, tested whether it is within the geographic regions of the reported origin, and then computed the mean accuracy per population. More specifically, we index our individual as the *j*th sample from the *i*th population that consists of *n*_*i*_ individuals. For all populations, excluding the individual in question, the average admixture proportions and geographical coordinates were calculated as ${\overline{\theta}}_m=\frac{\sum_s{\theta}_{m,s}}{n_m}$ where ${\overline{\theta}}_m$ is the parameter vector for the *s*th individual from the *m*th population, and *n*_*m*_ is the size of the *m*th population. For the *i*th population, the adjusted average will be ${\overline{\theta}}_i^{-j}=\frac{\sum_{l\ne j}{\theta}_{i,l}}{n_i-1}$. This procedure was repeated for each value of *K*.

A set of 245 genuine *M. truncatula* accessions with geographical coordinates (latitudes and longitudes) served as the reference set for ProvenancePredictor. Seventeen accessions, among which the Jemalong-A17 accession that is used as the reference genome [11], were of unknown origin and not included in the reference set.

### Computation of the eight-dimensional vector of admixture proportions for the *M. truncatula* accessions

To provide definitive population identification, the final admixture frequencies of the eight components for the 262 *M. truncatula* accessions were calculated by applying ADMIXTURE in the supervised mode. Accessions were then clustered into populations using hierarchical clustering based on their genome admixture proportions, using Euclidean distance and the “average” link. Relationships among accessions were based on genetic distances computed from the 840 K SNP dataset (R package SNPRelate), and a dendrogram was computed and drawn using R packages ape and geiger.

Maps and sample locations were drawn using the rworldmap, rgdal, mapplots, and maptools R packages.

### Phenotypic characterization of quantitative resistance to *Verticillium alfalfae* in *M. truncatula*

A set of 313 accessions of *M. truncatula* has been assessed for their response to Verticillium wilt, including 242 already sequenced accessions from the HapMap project [109]. *M. truncatula* seeds were from our own collection or obtained from the INRA *Medicago truncatula* Stock Center (Montpellier, France). All the *M. truncatula* accessions have been phenotyped using an augmented randomized block design in three independent replicates for the already sequenced (reference) accessions and two replicates for the other accessions. Between four and ten plants per genotype were used in each replicate. Ten-day-old plants were root inoculated as described in Ben et al. [82]. Disease development was monitored for 32 days two or three times a week and rated using a scale from “0” (no symptoms) to “4” (dead plants). At the end of the experiment, the maximum symptom score (MSS) was obtained for each plant. The LS mean of the MSS for each accession was calculated using the linear model *y*_*ijk*_ = *μ* + block_*i*_ + accession_*j*_ + *ϵ*_*ijk*_ (*y*_*ijk*_ the maximum disease score for the *k*th plant of the *j*th accession of the *i*th block; *ϵ*_*ijk*_, the residual) using R.

### Relationship between admixture proportions and quantitative phenotypic variables

The relationships between genome components and phenotypes were estimated using linear models. Because of dependencies among the predictors (the proportions of genome components must sum to one), a systematic search for the best minimum model was done using the leaps R package or use of the step function with both directions, employing a significance level of *α* = 5 % as the benchmark for using a predictor.

The 19 WorldClim bio-climatic variables (30 s resolution, downloaded at http://www.worldclim.org/current) were extracted for each accession’s location, using the reported latitude and longitude for that accession (raster R package). For each accession, the admixture components are the fractions that each of the eight sub-populations contributes to the accession’s genome. Thus, we have an Nx8 numeric matrix, assuming there are N accessions. Then, each of the eight sub-populations (i.e., admixture components in the text) is represented by an *N*-dimensional vector. On the other hand, each bio-climate variable was also represented by an *N*-dimensional vector as the bio-climate variables were extracted at each accession’s location. So, the correlation can be calculated. The relationships between genome components, the 19 WorldClim bio-climatic variables, and geography (latitude, longitude, and altitude) were modeled using redundancy analysis (RDA). RDA of admixture proportions with bio-climatic variables conditional to geography was also computed to estimate effects of climate “corrected for” the geography. Total inertia explained by the RDA model was partitioned among geography and climate, separately or combined. The RDA was computed using the vegan R package.

Spatial interpolation of phenotypic traits was performed using a thin plate spline method, with a smoothing parameter of *λ* = 0.005, as implemented in the R package fields.

### Genomic selection algorithms and prediction reliabilities

Genomic selection models were computed based on the 840 K SNP dataset. For all phenotypes, ridge regression best linear unbiased predictor (RR-BLUP), kinship-BLUP (G-BLUP), BayesB, reproducing kernel Hilbert space (RKHS), and least absolute shrinkage and selection operator (LASSO) regression were computed using the rrBLUP [74], BGLR [111], and glmnet [112] R packages.

We optimized the training sets by stratified sampling [61], that is the training sets are created by selecting a number of genotypes from each population proportional to the size of the population. Consequently, populations with more accessions will have a larger representation in the training set than smaller clusters. Fifty rounds of fivefold cross-validation were used to compute reliabilities of the GS and WhoGEM models. Briefly, at each round, the dataset is split into five non-overlapping subsets. Genotypes and phenotypes of the accessions of four subsets are used to compute the model (the training set). The predicted values of phenotypes are computed for the remaining subset (the test set). Correlations between the predicted and observed values of the test set are a measure of the model’s reliability.

Unless otherwise stated, all computations were done using the R statistical environment [113].

## Additional files

Additional file 1: **Figure S1.** Geographical location of the 245 *Medicago truncatula* accessions used in this analysis. **Figure S2.** ADMIXTURE proportions for 262 *Medicago truncatula* accessions, by increasing putative *K*. **Figure S3.** Bayesian information criteria (BIC) as a function of increasing values of *K*, using discriminant analysis of principal components (DAPC) applied on the 840 K SNP dataset for 262 *M. truncatula* accessions. **Figure S4.** ProvenancePredictor indicates *K* = 8 as the first minimum number of admixture components to minimize the median distance between predicted and recorded location (left scale) and maximize correct assignment to country of origin (right scale). **Figure S5.** Relation between geographical and genetic distances among 245 *M. truncatula* accessions with known location, for *K* = 8. **Figure S6.** Dendrogam of genetic relationships between the 262 *M. truncatula* accessions of the eight *M. truncatula* populations, based on analysis of the 840 K SNP dataset. **Figure S7.** Geographical repartition of phenotypic values for several quantitative functional traits in 226 *M. truncatula* accessions. **Figure S8.** Assessing *M. truncatula* initial sampling based on the relationship between admixture component and partial resistance to *V. alfalfae*. **Figure S9.** *M. truncatula* accessions repartition, with admixture components visualized as pies, within the Mediterranean Basin climatic zones following Köppen-Geiger climate classification. (PDF 11091 kb)

Additional file 2: **Table S1.** SNP selection process for admixture analysis. (PDF 48 kb)

Additional file 3: **Table S2.** Classification of 262 *M. truncatula* accessions in eight populations. (CSV 26 kb)

Additional file 4: **Table S3.** Maximum symptom score of 262 *M. truncatula* accessions in response to root infection by *V. alfalfae*. (CSV 6 kb)

Additional file 5: **Table S4.** Maximum symptom score of 71 previously uncharacterized *M. truncatula* accessions in response to root infection by *V. alfalfae*. (CSV 3 kb)

Additional file 6: **Table S5.** Mean comparisons for quantitative resistance, among groups of *M. truncatula* accessions. (PDF 73 kb)

Additional file 7: Code 1. R code for the ProvenancePredictor function (R (txt) (R 13 kb)

Additional file 8: Code 2. R code for the ProvenancePredictor cross-validation function (R (txt) (R 13 kb)
