# Supplementary material for: WhoGEM: an admixture-based prediction machine accurately predicts quantitative functional traits in plants
Source: Genome Biol. 2019 May 28;20:106. doi: 10.1186/s13059-019-1697-0 (PMC6537182; doi:10.1186/s13059-019-1697-0)
Supplement: Supplementary file 1 — Figure S1. Geographical location of the 245 Medicago truncatula accessions used in this analysis. Figure S2. ADMIXTURE proportions for 262 Medicago truncatula accessions, by increasing putative K. Figure S3. Bayesian information criteria (BIC) as a function of increasing values of K, using discriminant analysis of principal components (DAPC) applied on the 840 K SNP dataset for 262 M. truncatula accessions. Figure S4. ProvenancePredictor indicates K = 8 as the first minimum number of admixture components to minimize the median distance between predicted and recorded location (left scale) and maximize correct assignment to country of origin (right scale). Figure S5. Relation between geographical and genetic distances among 245 M. truncatula accessions with known location, for K = 8. Figure S6. Dendrogam of genetic relationships between the 262 M. truncatula accessions of the eight M. truncatula populations, based on analysis of the 840 K SNP dataset. Figure S7. Geographical repartition of phenotypic values for several quantitative functional traits in 226 M. truncatula accessions. Figure S8. Assessing M. truncatula initial sampling based on the relationship between admixture component and partial resistance to V. alfalfae. Figure S9. M. truncatula accessions repartition, with admixture components visualized as pies, within the Mediterranean Basin climatic zones following Köppen-Geiger climate classification. (PDF 11091 kb) [file 13059_2019_1697_MOESM1_ESM.pdf]

Fig. S1

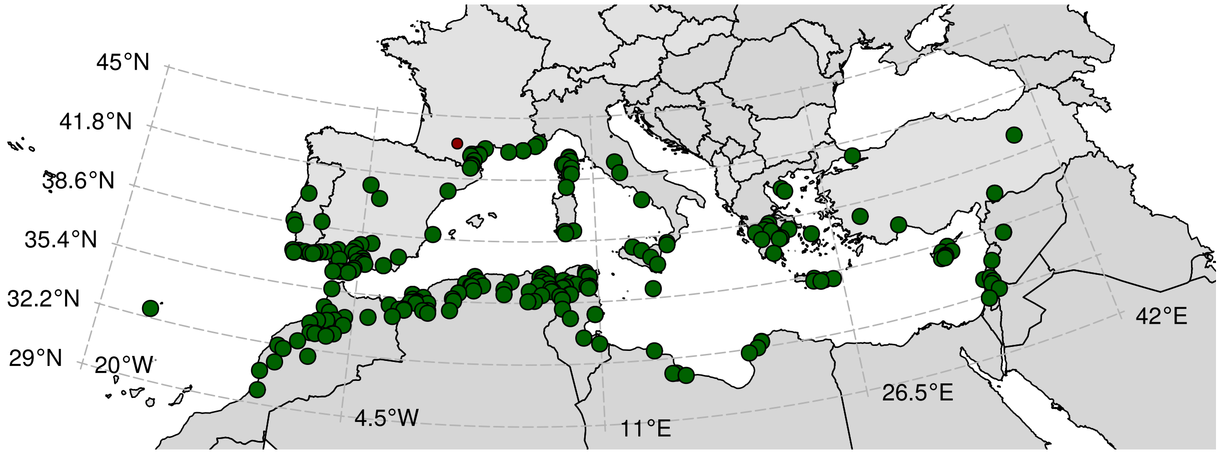

Fig. S1. Geographical location of the 245 *Medicago truncatula* accessions used in this analysis. 17 accessions without known spatial location were also included in the admixture analysis.
A Lambert Conical Conform projection (EPSG:3034), suitable for the Mediterranean Basin, was used to draw the geographical map.

Fig. S2

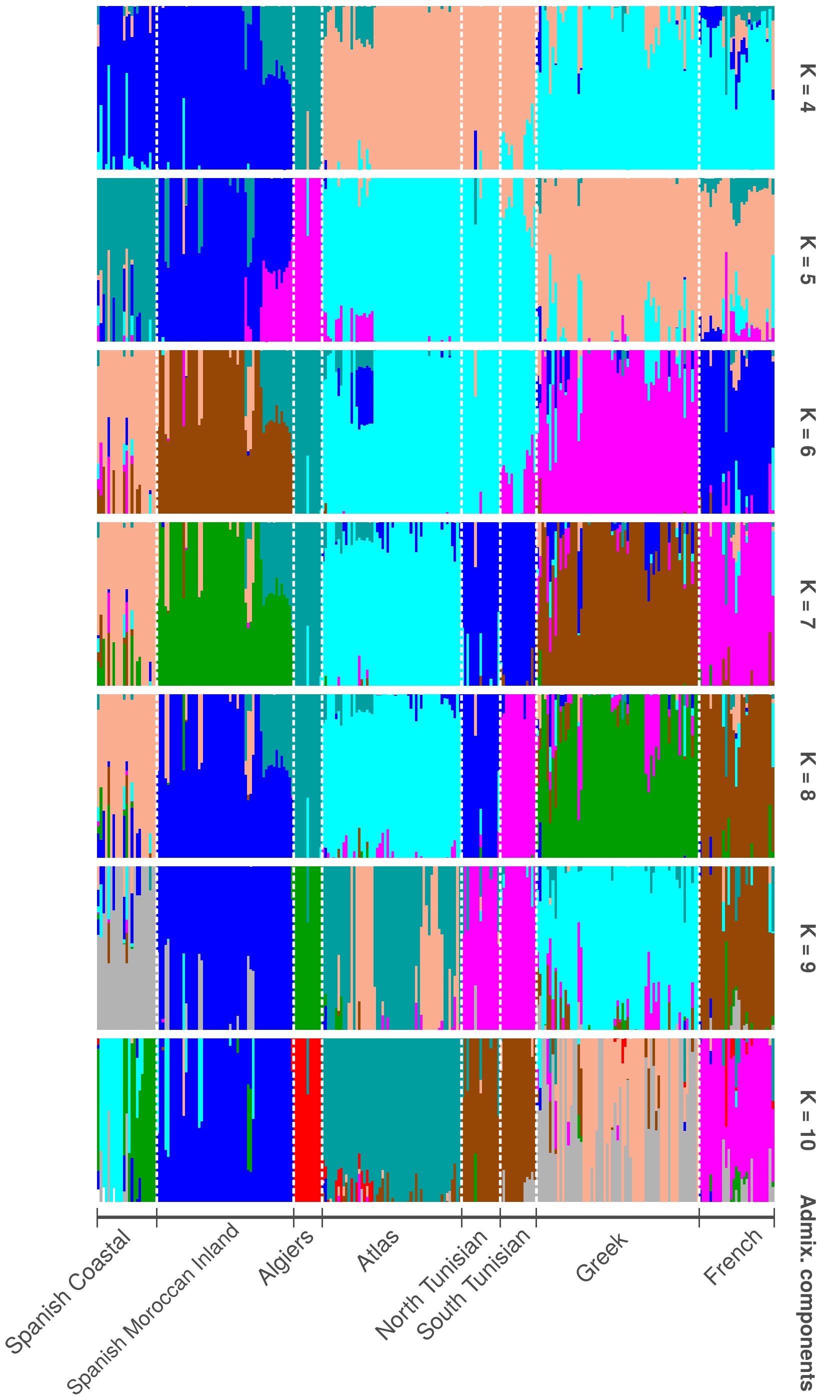

Fig. S2. `ADMIXTURE` proportions for 262 *Medicago truncatula* accessions, by increasing putative K. The X axis represents accessions sorted broadly according to their reported longitude and ancestry. Each accession is represented by a vertical stacked column of color-coded admixture proportions that reflects genetic contributions from putative ancestral populations. Population assignments based on $K = 8$ are reported for ease of comparison.

Fig. S3

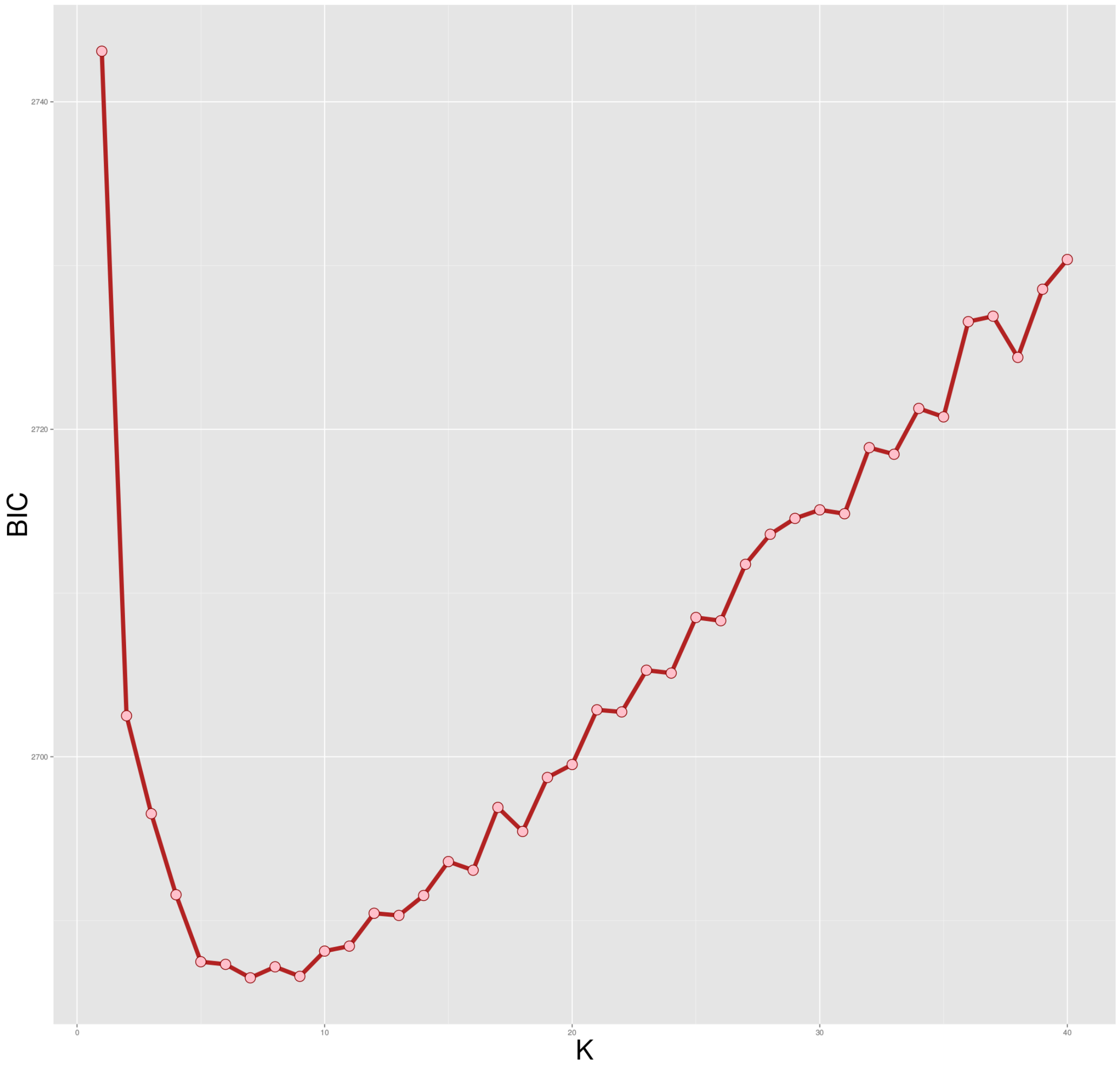

Fig. S3. Bayesian Information Criteria (BIC) as a function of increasing values of K, using Discriminant Analysis of Principal Components (DAPC) applied on the 840K SNP dataset for 262 *M. truncatula* accessions.

Fig. S4

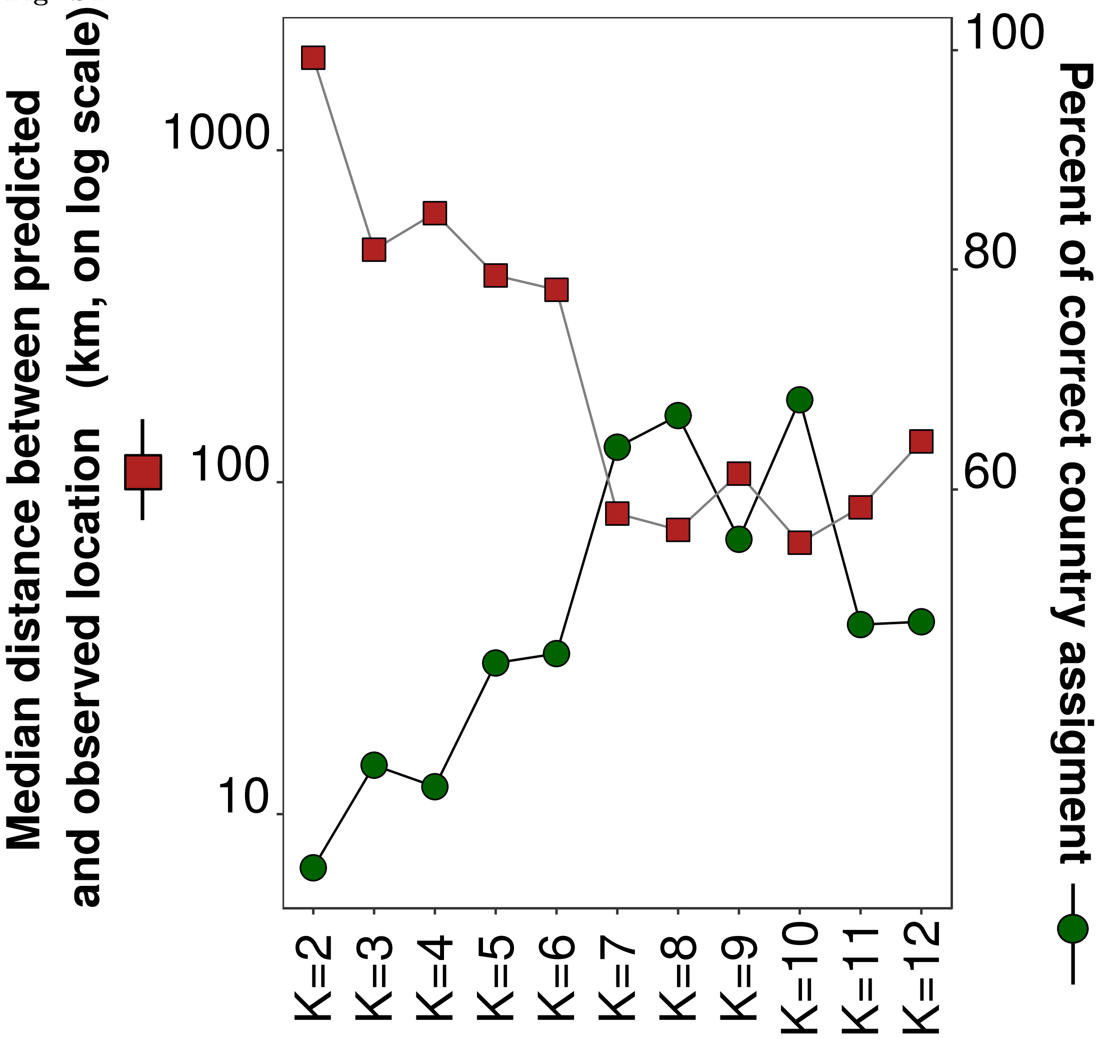

Fig. S4. ProvenancePredictor indicates $K = 8$ as the first minimum number of admixture components to minimize median distance between predicted and recorded location (left scale), and maximize correct assignment to country of origin (right scale).
Country is used as a surrogate for population of origin.

Fig. S5

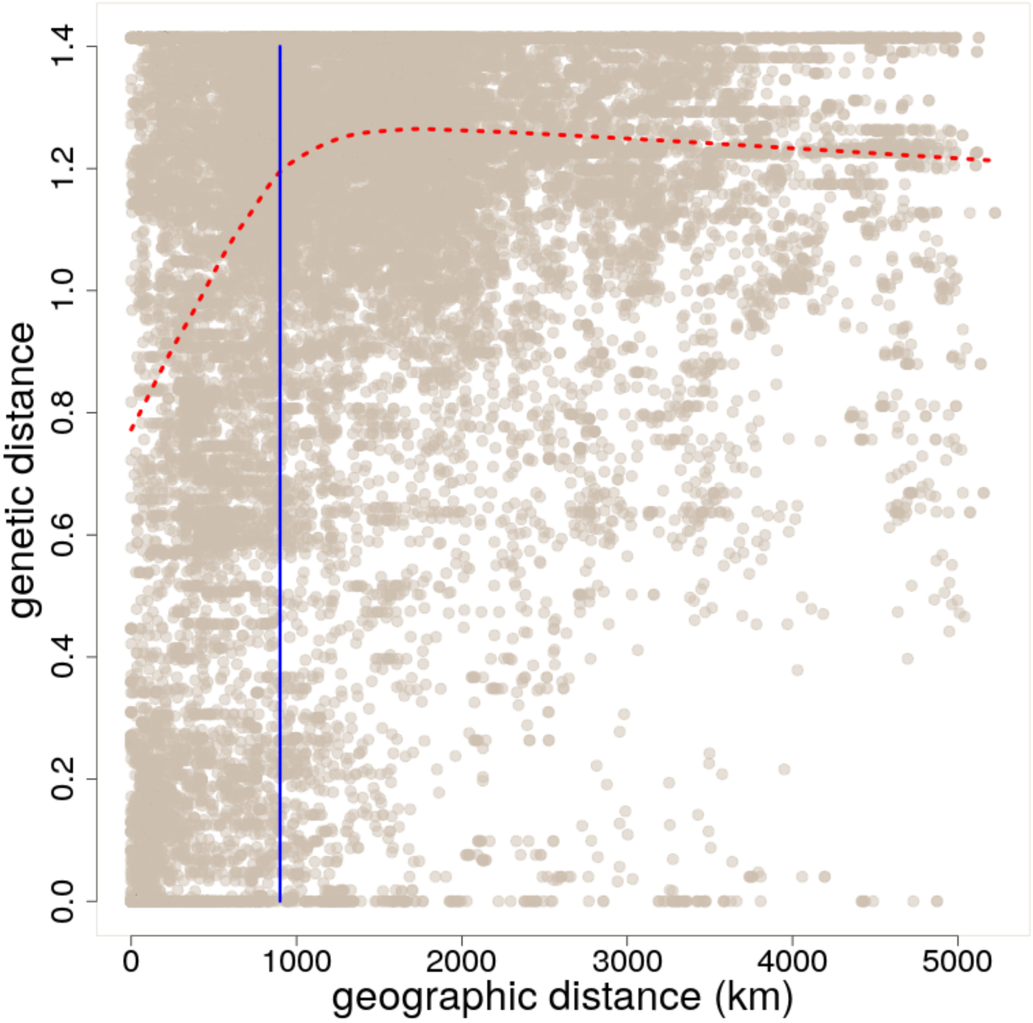

Fig. S5. Relation between geographical and genetic distances among 245 *M. truncatula* accessions with known location, for $K = 8$. The red dotted curve is a loess adjusted curve. The vertical blue line is drawn at 950km.

Fig. S6

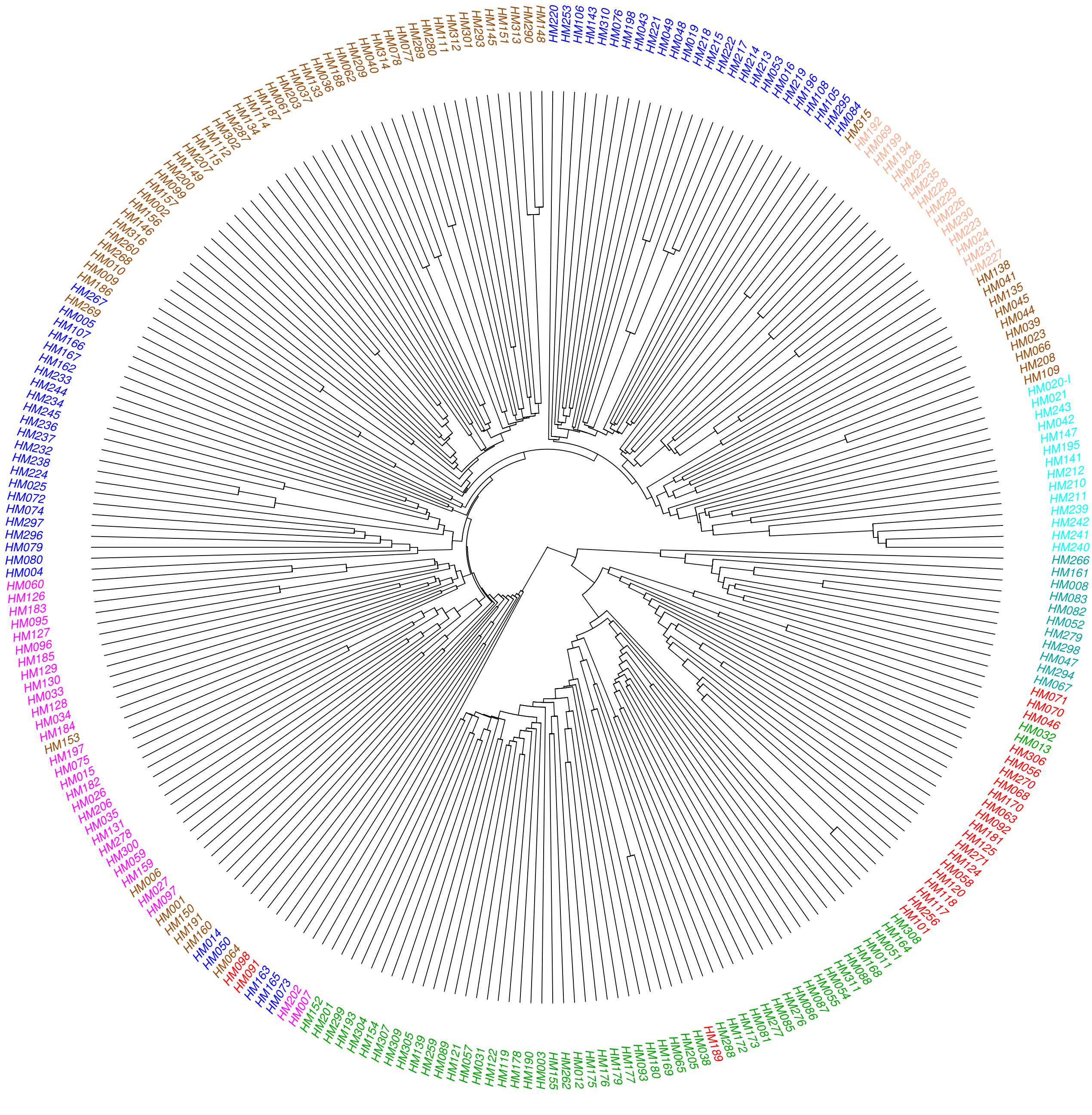

Figure S6. Dendrogam of genetic relationships between the 262 *M. truncatula* accessions of the eight *M. truncatula* populations, based on analysis of the 840K SNP dataset.

Fig. S7

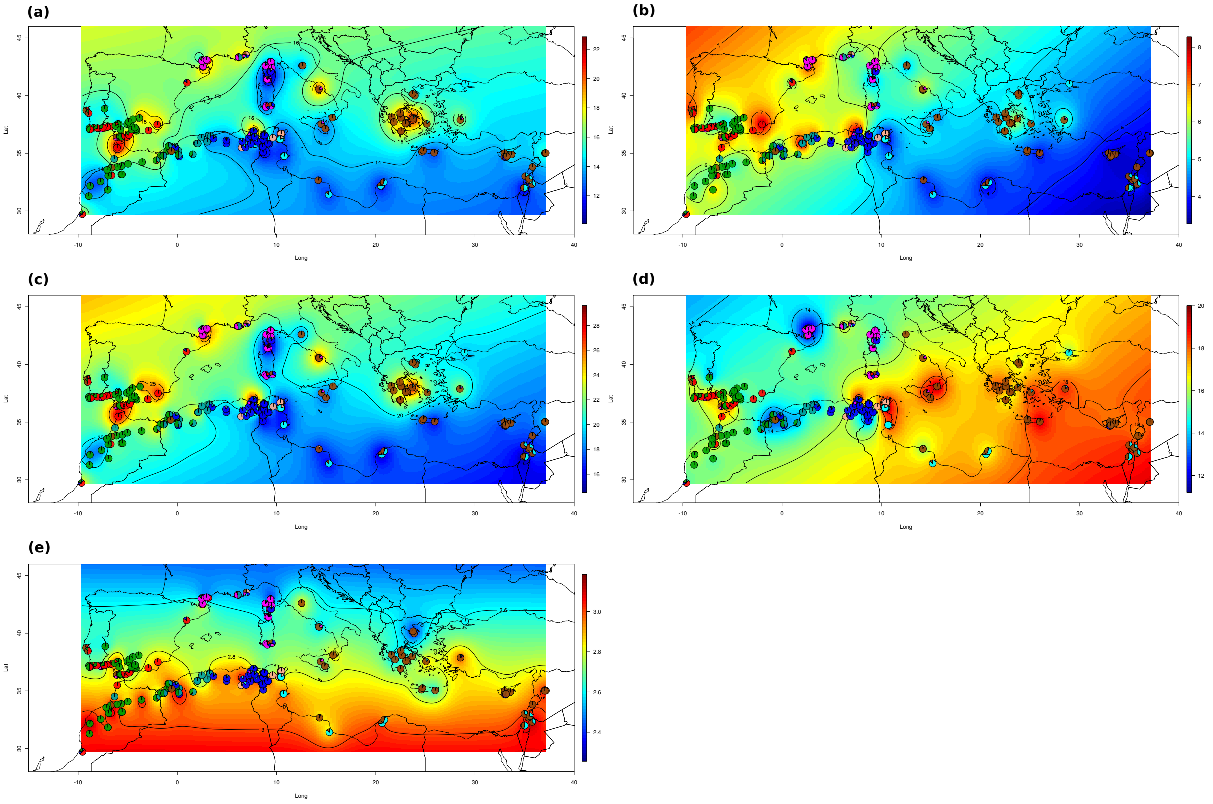

Fig. S7. Geographical repartition of phenotypic values for several quantitative functional traits in 226 *M. truncatula* accessions.

(a) number of nodules below 5 cm of root growth. (b) number of nodules in top 5 cm of roots. (c) total number of nodules. (d) final plant height before harvest (e) number of leaves at about two weeks.

For all traits, the scale is displayed as a color gradient over the geographical map, from lower values (blue) to higher values (red). Raw data from Stanton-Geddes *et al.* (2013)

Fig. S8

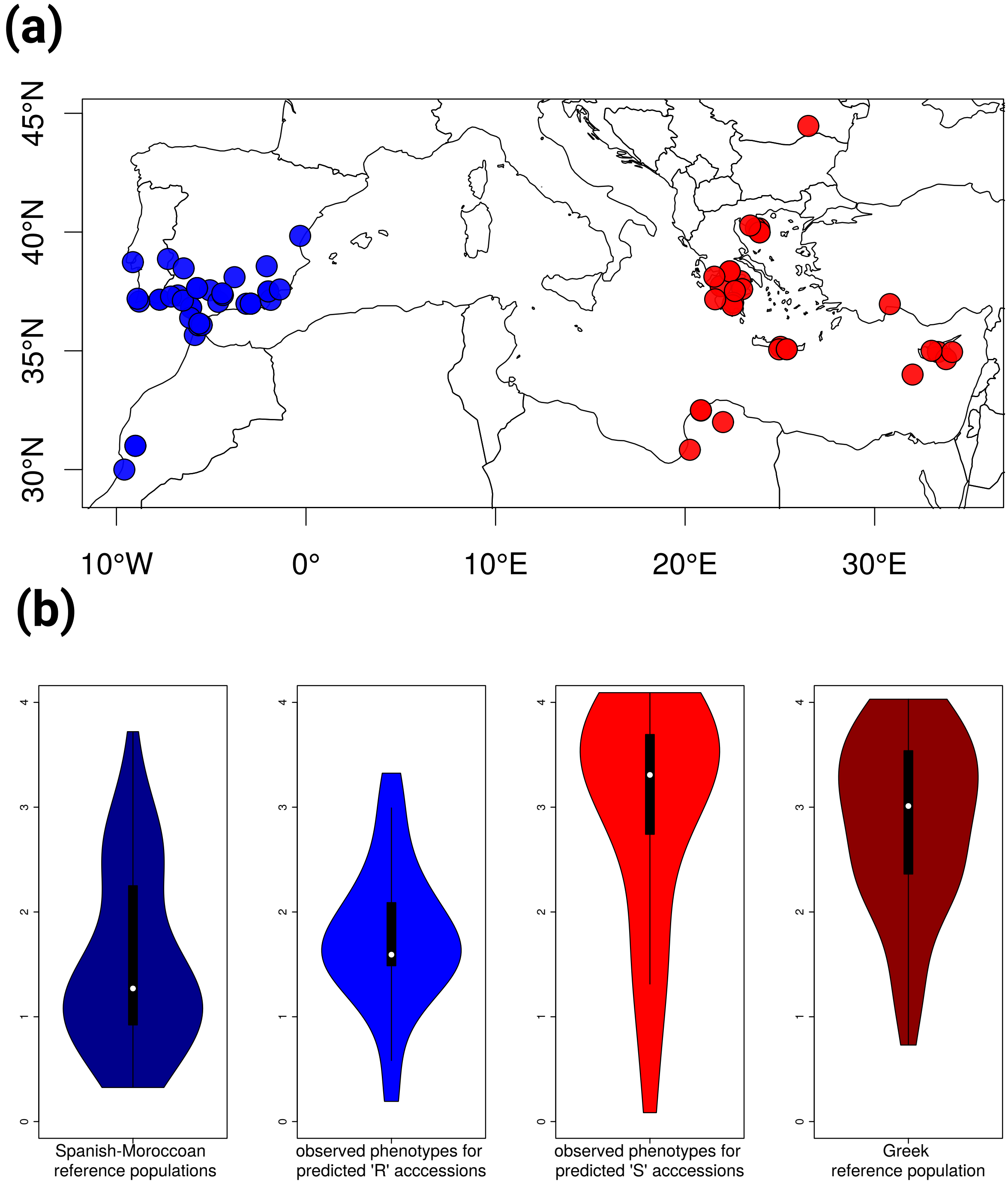

Fig. S8. Assessing *M. truncatula* initial sampling based on the relationship between admixture component and partial resistance to *V. alfalfae*.

(a) Sampling of new accessions in the geographic zone of the "Spanish Coastal" and "Spanish-Moroccan Inland' resistant populations, and in the geographic zone of the 'Greek' susceptible populations.

(b) Violin plots of Maximum Symptom Score (MSS) for reference and sampled accessions. Reference resistant accessions are sequenced accessions belonging to the "Spanish Coastal" and "Spanish-Moroccan Inland" populations, reference susceptible accessions are sequenced accessions belonging to the "Greek" population.

Fig. S9

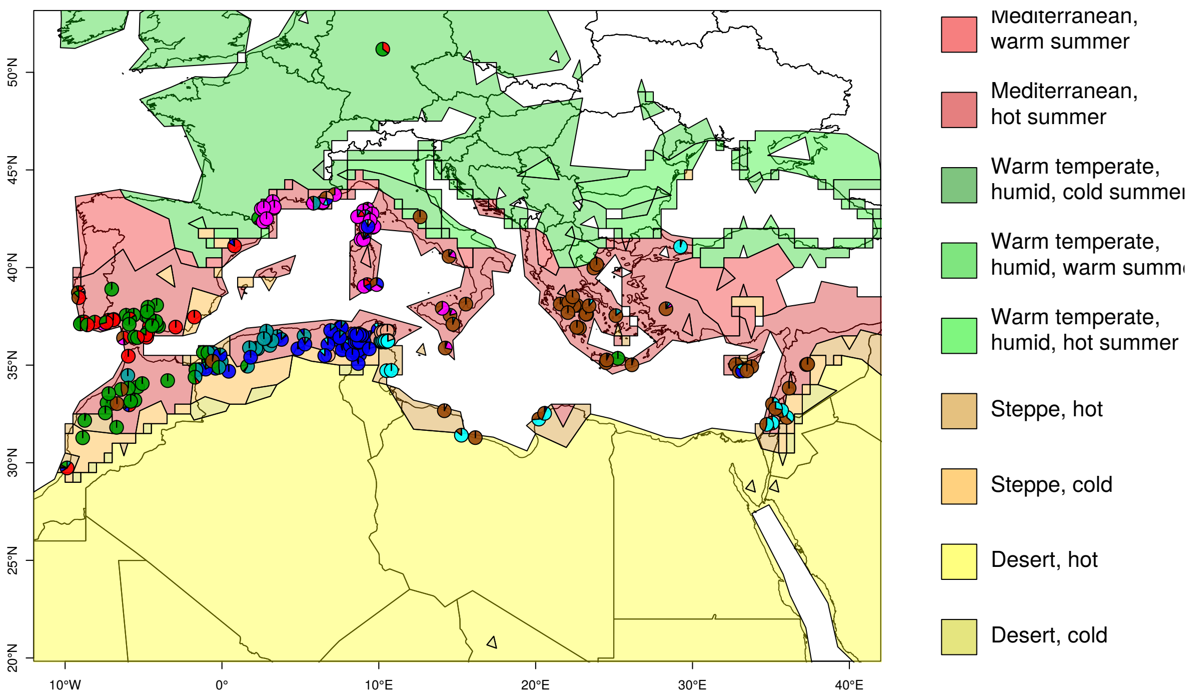

Fig. S9. *M. truncatula* accessions repartition, with admixture components visualized as pies, within Mediterranean Basin climatic zones following Köppen-Geiger climate classification.
