# Supplementary material for: WhoGEM: an admixture-based prediction machine accurately predicts quantitative functional traits in plants
Source: Genome Biol. 2019 May 28;20:106. doi: 10.1186/s13059-019-1697-0 (PMC6537182; doi:10.1186/s13059-019-1697-0)
Supplement: Supplementary file 2 — Table S1. SNP selection process for admixture analysis. (PDF 48 kb) [file 13059_2019_1697_MOESM2_ESM.pdf]

Table S1: SNP selection process for admixture analysis.

Chromosome	Avail. SNPs	QC SNPs	LD-pruned SNPs
Chrom. 1	4978489	158858	116038
Chrom. 2	4331584	136735	100011
Chrom. 3	5424868	165394	121174
Chrom. 4	5414544	170726	123580
Chrom. 5	4496375	133727	98578
Chrom. 6	3650081	97328	72271
Chrom. 7	4761519	151101	111584
Chrom. 8	4377934	135847	99935
total	37435394	1149716	843171

Quality Checked (QC) SNPs : genotyping rate $\geq$ 0.95 and Minor Allele Frequency $\geq$ 0.01. LD-pruned SNPs : Variance Inflation Factor $\leq$ 1.22 in a window of 300 SNPs.
