# Supplementary material for: WhoGEM: an admixture-based prediction machine accurately predicts quantitative functional traits in plants
Source: Genome Biol. 2019 May 28;20:106. doi: 10.1186/s13059-019-1697-0 (PMC6537182; doi:10.1186/s13059-019-1697-0)
Supplement: Supplementary file 6 — Table S5. Mean comparisons for quantitative resistance, among groups of M. truncatula accessions. (PDF 73 kb) [file 13059_2019_1697_MOESM6_ESM.pdf]

Supplementary Table 5: Mean comparisons for quantitative resistance, among groups of *M. truncatula* reference accessions of the two "Spanish" populations, unknown accessions sampled in Spain, reference accessions of the "Greek" population and unknown accessions sampled around Greece

	diff	lwr	upr	p.adj
Reference R vs Reference S	1.3286	0.9035	1.7537	0.0000
Predicted R vs Predicted S	1.2294	0.7070	1.7518	0.0000
Reference R vs Predicted R	-0.2308	-0.6859	0.2243	0.5544
Reference S vs Predicted S	-0.1316	-0.6280	0.3649	0.9018
Reference S vs Predicted R	1.0978	0.6211	1.5745	0.0000
Reference R vs Predicted S	-1.4602	-1.9360	-0.9844	0.0000

Reference: accessions with known admixture proportions ("Spanish Coastal", "Spanish Moroccan Inland" and "Greek" populations); Predicted: accessions with unknown admixture proportions sampled in Spain or Greece;
R: quantitative resistance; S: susceptibility.

MSS values of those accessions expected to be resistant or susceptible differ significantly (ANOVA $P < 2 \cdot 10^{-16}$), and are not significantly different from the initial sampling.
